# Investigation of the Mechanisms of Cytotoxic Activity of 1,3-Disubstituted Thiourea Derivatives

**DOI:** 10.3390/ph14111097

**Published:** 2021-10-28

**Authors:** Paulina Strzyga-Łach, Alicja Chrzanowska, Katarzyna Podsadni, Anna Bielenica

**Affiliations:** Chair and Department of Biochemistry, Medical University of Warsaw, 02-097 Warszawa, Poland; pstrzyga@wum.edu.pl (P.S.-Ł.); achrzanowska@wum.edu.pl (A.C.); kpodsadni@wum.edu.pl (K.P.)

**Keywords:** thiourea, cytotoxic activity, apoptosis, interleukin-6, trypan blue assay

## Abstract

Substituted thiourea derivatives possess confirmed cytotoxic activity towards cancer but also normal cells. To develop new selective antitumor agents, a series of 3-(trifluoromethyl)phenylthiourea analogs were synthesized, and their cytotoxicity was evaluated in vitro against the cell line panel. Compounds **1**–**5**, **8**, and **9** were highly cytotoxic against human colon (SW480, SW620) and prostate (PC3) cancer cells, and leukemia K-562 cell lines (IC_50_ ≤ 10 µM), with favorable selectivity over normal HaCaT cells. The derivatives exerted better growth inhibitory profiles towards selected tumor cells than the reference cisplatin. Compounds incorporating 3,4-dichloro- (**2**) and 4-CF_3_-phenyl (**8**) substituents displayed the highest activity (IC_50_ from 1.5 to 8.9 µM). The mechanisms of cytotoxic action of the most effective thioureas **1**–**3**, **8**, and **9** were studied, including the trypan blue exclusion test of cell viability, interleukin-6, and apoptosis assessments. Compounds reduced all cancerous cell numbers (especially SW480 and SW620) by 20–93%. Derivatives **2** and **8** diminished the viability of SW620 cells by 45–58%. Thioureas **1**, **2**, and **8** exerted strong pro-apoptotic activity. Compound **2** induced late apoptosis in both colon cancer cell lines (95–99%) and in K-562 cells (73%). All derivatives acted as inhibitors of IL-6 levels in both SW480 and SW620 cells, decreasing its secretion by 23–63%.

## 1. Introduction

Cancer is now considered as the second cause of death after cardiovascular disorders. It is estimated that the number of newly diagnosed tumor cases will increase to 15 million episodes every year [1]. Currently, the most common method used for the treatment of cancer is chemotherapy. However, powerful chemotherapeutics also have an adverse impact on non-cancerous cells, slowing their growth and/or inducing apoptosis. Thus, the main challenge for the pharmaceutical industry is to synthesize new anticancer agents that are more effective and selective but less toxic for normal cells.

**Figure 1:**
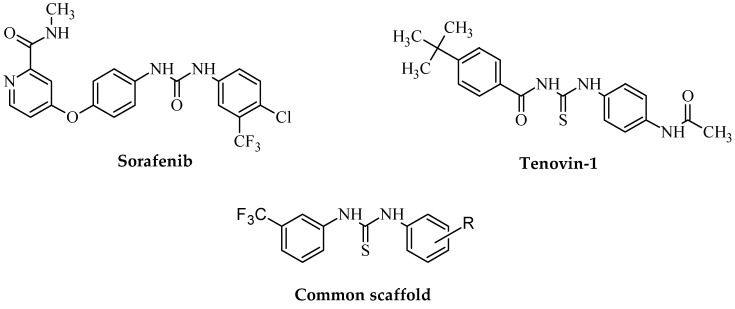
Anticancer agents derived from diaryl(thio)urea and the common scaffold of the designed target 3-(trifluoromethyl)phenylthioureas.

One of the most suitable strategies in the field of drug development is the combination of two bioactive nuclei. The (thio)urea branch is an element of several medicines with anticancer profiles, such as sorafenib, multikinase-inhibitory diarylthiorea derivative, or tenovin-1, the benzylthiourea, which acts as a reversible inhibitor of class III HDAC sirtuins (Figure 1). On the other hand, it was reported that (hetero)aryl terminal fragments of thiourea moiety, enriched with electron-negative substituents, could provide biological responses, not only cytotoxic [2,3,4], but also antibacterial [2,3,4,5,6,7,8], antiviral [2,9,10,11,12], antimycobacterial [5,13], antioxidant [14], and anti-inflammatory [6] properties, as well as central nervous system activation [15,16,17,18]. 

Large numbers of 1,3-disubstituted derivatives of urea and thiourea have been reported to possess antiproliferative properties against various solid and leukemia tumor cell lines, simultaneously resulting in low side effects. The most effective agents were found in a group of derivatives with electron-withdrawing substituents introduced to the terminal phenyl rings. In recent years, some trifluoromethyl- and trifluoromethoxyphenyl(thio)ureas bearing the thiochroman ring have been synthesized, which exerted an ovarian cancer cell inhibitory effect [19]. Several biphenyl thiourea derivatives, which incorporated CF_3_, nitro, and halogen groups on the pendent aryl rings, were described as inhibitors of lung cancer cell A549 growth, with blocking of K-Ras protein as the identified mechanism [20]. It was reported that 2-bromo-5-(trifluoromethoxy)phenylthiourea, the derivative of quinazoline, suppressed proliferation and migration of human cervical HeLa cells via inhibition of the Wnt/β-catenin signaling pathway [21]. Representative 7-trifluoromethyl-quinolinyl-piperazine compounds based on the thiourea scaffold showed improved anti-breast cancer action [22]. In their presence, the membrane integrity of the cytoplasm, mitochondria, and lysosomes of cancer cells were compromised. Series of 4-thiazolidinone-phenylaminopyrimidine hybrids bearing *orto*-chloro and *para*-CF_3_ substituents displayed anticancer activity on chronic myeloid leukemia cells, inducing programmed cell death by inhibition of AbI kinase [23]. Within a group of 1,2,4-triazole-linked (thio)urea conjugates synthesized by Tokala et al., the 4-cyanophenyl derivative with bis(trifluoromethyl)phenyl moiety expressed the highest apoptosis-inducing activity against the breast cancer cell line [24]. The representative diarylurea endowed with both CF_3_/OCF_3_ substituents has recently been developed as an inhibitor of the most lethal and aggressive subtype of breast cancer [25]. Selective kinase inhibitory agents towards hepatocellular carcinoma cells were found among a series of conformationally restricted fluorinated ureas, analogues of sorafenib [26].

Within the thiourea derivatives, halogenated phenyl-containing heterocyclic thioureas play important roles as anticancer agents against solid tumors, such as derivatives of 1,3,4-thiadiazine [27], dihydroquinoline [28], pyridine [29], piperidine [30], quinazoline [31,32], or thiazole [33]. Their mechanisms of action include inhibition of vascular endothelial growth factor receptor 2 [27], epidermal growth factor receptor kinase [31,32], or acetylcholinesterase [33]. Similarly, the presence of the nitrophenyl moiety at the thiourea branch is identified to impart promising cytotoxic activity towards various solids tumors, including lung, colorectal [34], prostate, and breast [35] carcinoma, acting via mitogen kinase enzyme (MK-2) inhibition [34].

The 3-(Trifluorometyl)phenylthiourea moiety is a versatile scaffold in medicinal chemistry, also used previously by our team for the design of new compounds with variable and improved pharmacological profiles, mainly antimicrobial [2,8], antiviral [2,12], and CNS-activating [15] compounds. Herein, we focus on possible mechanisms of the cytotoxic properties of a series of 3-(trifluorometyl)phenylthiourea analogs, incorporating differential electron-withdrawing terminal groups.

## 2. Results and Discussion

### 2.1. Chemistry

**Scheme 1:**
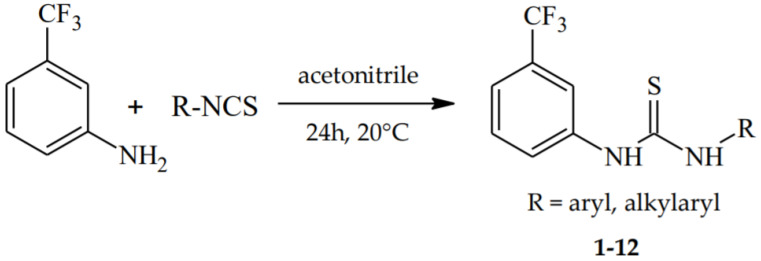
Synthetic procedure for 3-(trifluoromethyl)phenylthiourea derivatives **1**–**12**.

The final 1,3-disubstituted thioureas **1**–**12** were synthesized in a single-step reaction of 3-(trifluoromethyl)aniline with various isothiocyanates, belonging to a group of dihalogenophenyl (**1**–**4**), halogenomethylphenyl (**5**, **6**), alkylphenyl (**12**), or monophenyl-substituted (**7**–**11**) derivatives (Scheme 1). The presented selection of the phenyl ring terminal groups allowed an investigation of the impact of the substitution isomerism, as well as the influence of the electron-withdrawing elements attached to the benzene ring on the biological properties within the tested thiourea series. The synthesis of compounds **1**, **2**, **9**, **10**, and **12** was described previously. The structures of the newly synthesized derivatives (**3**–**8**, **11**) were characterized by both ^1^H and ^13^C NMR spectroscopy and HRMS analysis.

### 2.2. Biological Studies

#### 2.2.1. Cytotoxic Activity

As a first step in assessing their cytotoxic properties, all thioureas of the series were assayed against four human carcinoma cell lines, such as SW480 (primary colon cancer), SW620 (metastatic colon cancer), PC3 (metastatic prostate cancer), and K-562 (chronic myelogenous leukemia), as well as against the normal cell line HaCaT (immortalized human keratinocytes). Table 1 lists the compound concentrations that produced 50% of growth inhibition (IC_50_, μM), generated by the MTT method [36], in comparison with two commonly used chemotherapeutic agents, doxorubicin and cisplatin.

Dihalogenophenyl derivatives (**1**–**4**), followed by *para*-substituted thioureas (**8**, **9**) were the most active among the series towards all tumor cell lines. They were particularly potent against SW620 cells, which appeared to be the most susceptible among the studied pathological cells. The lowest IC_50_ was achieved by 3,4-dichlorophenylthiourea (**2**) and equaled 1.5 ± 0.72 μM. Its isomer **3**, and also derivatives of 4-(trifluoromethyl)phenylthiourea (**8**) and 4-chlorophenylthiourea (**9**), inhibited the growth of metastatic colon cancer cells at concentrations ranging from 5.8 ± 0.76 to 7.6 ± 1.75 μM. The 3-chloro-4-fluorophenylthiourea (**1**) filled up the group of the most outstanding cytotoxic agents towards the SW620 cell line (IC_50_ = 9.4 ± 1.85 μM). Moderate anticancer potency at the level of 14.0–18.7 μM was observed for derivatives **4**, **10**, and **11**. Importantly, the strongest inhibitors of the growth of these cells (**1**–**4**, **8**, **9**) were also described by high selectivity indexes (SIs), ranging from 4.6 (compound **1**) to 16.5 (compound **2**). In addition, when compared to cisplatin, substances **2**, **3**, and **9** were found to be more effective, considering both their IC_50_ values and selectivity factors. The potency of compound **2** was up to 4.5 times stronger and its SI and 18 times higher than the reference metalodrug.

The disubstituted chlorine-containing derivatives **2** and **5**, as well as 4-(trifluoromethyl)phenyl compound (**8**), applied at concentrations of 7.3–9.0 μM were able to effectively inhibit primary SW480 cell lines, while also being more potent than cisplatin. Additionally, within all compounds, the thiourea **5** was highly selective against SW480 cells vs. the other pathological lines tested. On the other hand, the selectivity of both thioureas towards HaCaT cells was advantageous, extending between 2.7 and 7.6. Moreover, the values of IC_50_ of their close structural analogs, **1** and **6**, ranged from 12.7 ± 1.53 to 15.6 ± 4.10 μM.

A significant cytotoxic effect on the erythroleukemic K-562 cell lines was observed for dihalogenophenylthioureas **1** and **2**, as well as for the monosubstituted derivative **9**, with all of them containing at least one chlorine atom attached to the terminal ring. Analogs **1** and **2** were 20–30% more effective and several-fold more selective than the reference drug cisplatin. The antiproliferative potency of the *para*-substituted derivatives **9** and **10** was estimated at IC_50_ of 10.2–12.9 μM.

Prostate cancer cells belonged in the group that were the least vulnerable to the presence of thiourea compounds; however, three halogenated analogs still exerted higher than (compounds **4**, **8**) or comparable (derivative **3**) activity to cisplatin. Their concentrations corresponding to 50% growth inhibition of the PC3 line varied from 6.9 ± 1.64 to 13.7 ± 7.04 μM, and their selectivity indexes were also favorable (1.3–6.0). The most potent 4-(trifluoromethyl)phenylthiourea (**8**) was also strongly effective against both colon cancer lines but not K-562 cells.

The cytotoxic action of the 2-phenylethylthiourea derivative **12** against cancer cells differed from 23.8 ± 0.45 to 38.2 ± 3.10 μM, depending on the tumor line tested, and it was the only inefficient compound of the designed series. It is worth mentioning that generally the lower the IC_50_ values assigned, the higher the SI found. The most promising drug candidates (**1**–**4**, **8**, **9**) were weakly cytotoxic towards normal HaCaT cell lines. While none of the tested compounds were as potent as ciprofloxacin, the most effective of them expressed a better cytotoxic profile than cisplatin, and possessed higher selectivity indexes in comparison with both referential chemotherapeutics.

A wide selection of the character, location, and number of phenyl ring substituents of the thiourea branch allowed investigation of the influence of the structure of the studied compounds on their antitumor activity. According to our studies, the terminal benzene moiety functionalities were arranged with their increasing impact on cytotoxicity as follows: 4-bromo- (**11**) < 2-(trifluoromethyl)- (**7**) < 2-methyl-5-chloro- (**6**) < 4-cyano- (**10**) < 2-methyl-3-chloro- (**5**) < 2,3-dichloro- (**4**) << 2,4-dichloro- (**3**) < 4-chloro- (**9**) < 3-chloro-4-fluoro- (**1**) < 4-(trifluoromethyl)- (**8**) < 3,4-dichloro- (**2**). As shown, the most pronounced cytotoxic effect was associated with an incorporation of two halogen atoms in the benzene ring, whereby chlorine (**2**, **3**) or fluorine (**1**) were in the *para-* position. Considering the active monosubstituted derivatives, this position of the phenyl moiety was favored by the trifluoromethyl group (**8**) or chlorine (**9**). The replacement chlorine with fluorine is less beneficial in the case of disubstituted derivatives (**2** → **1**) and heavily unfavorable in a group of monosubstituted thioureas (**9** → **11**). Similarly, the noticeable decrease in bioactivity was observed when the electron-donating methyl group was introduced instead of the second halogen (**5**, **6**). Furthermore, from the closest mutual arrangement of both methyl and chlorine groups with the highest activity, the thiourea **5** is more effective against SW480 and selective towards HaCaT cells than its isomer **6**. Considering other isomers of the substituent position, the most cytotoxic profile was exerted by compound **2** with the 3,4-dichlorophenyl fragment. The change of substituent locations to carbons 2,4 or 2,3 (derivatives **3** and **4**, respectively) led to a gradual reduction in their biological potency. By analogy, the replacement of the *ortho*-substituted CF_3_ group (**7**) for the *para* (**8**) position was much more fruitful. While an exchange of this voluminous substituent for chlorine (**8** → **9**) still gave a strongly active analog, switching it into the cyano (**10**) or bromo (**11**) group considerably diminished the antitumor effect. Finally, the introduction of the unsubstituted alkylphenyl group (**12**) to the thiourea branch dramatically decreased the compound’s bioactivity.

The most potent derivatives (**1**–**3**, **8**, **9**) were selected for further investigations of their mechanisms of cytotoxic action.

#### 2.2.2. Antiproliferative Activity

In order to estimate the tumor and normal cell population density and their viability after treatment with compounds **1**–**3**, **8**, and **9**, the trypan blue dye exclusion assay was performed. The live cell number of all cancerous cells incubated for 72 h with the studied thioureas was considerably lower in comparison with the controls (Table 2; Figure S1A). The highest reduction in cell amount was denoted for both colon SW480 and SW620 cells treated with the dichlorophenyl derivative **2**, and it accounted for 93%. This compound also considerably reduced the PC3 and K-562 cell number by 69% and 66%, respectively. Similarly, a noticeable decline in both colon cancer cell populations was observed after treatment with the *ortho*-substituted compound **8**. The number of live cells equaled 12% and 18%, respectively, as compared to controls. This thiourea also led to a reduction of PC3 cells of 38%, and leukemia K-562 cell lines of 27%. The number of SW620 and PC3 cells decreased by 72% and 63% in the presence of *para*-substituted compound **9**. However, the reducing influence of this thiourea on the number of other cancerous cells was at the level of 21–32%. It is worth noting that the amount of PC3 cells also diminished to 24% compared to the control after long-term exposure to the IC_50_ concentration of dihalogenophenyl derivative **1**. A significant effect on the other studied pathological cells was also noticed and accounted for 32–46%. Furthermore, the derivative **3** reduced the amount of live cancer cells by 32–59%, of which its decreasing impact on colon cancer cells was the greatest. The obtained results show that thioureas **1**–**3**, **8**, and **9** exerted a cytostatic effect on cancer cells, suppressing their growth and proliferation.

In addition, after thiourea treatment, the viability of the majority of pathological cell lines was diminished, which proved not only the cytostatic, but also the cytotoxic influence of the selected derivatives (Table 2; Figure S1B). This effect was clearly observed in both SW480 and SW620 cells for thioureas **1**, **2**, and **9**. The largest decrease of cell viability was found for the compound **2** (by 48% and 58%, respectively), while substances **2** and **9** were efficient in 16–24%. Additionally, the derivative **8** reduced the viability of SW620 cells by 45%. Derivatives **2**, **3**, and **9** decreased the survival of PC3 cells by 15–39%, as compared to the control, and the most evident cytotoxic activity in these cells was observed for **3**. The K-562 cell line was the least sensitive to incubation with the evaluated compounds. Its viability was diminished by 10–15% after 72 h of contact with thioureas **2**, **3**, and **8**. Importantly, the various concentrations of the target compounds did not affect normal HaCaT cells’ viability.

#### 2.2.3. Apoptotic Activity

**Figure 2:**
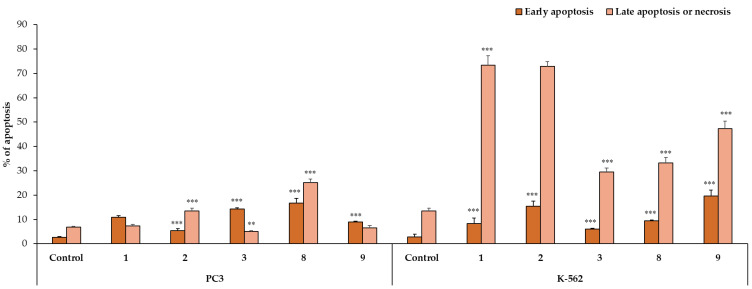
The effect of compounds **1**–**3**, **8**, and **9** on early and late apoptosis in SW480, SW620, and HaCaT cells. Cells were incubated for 72 h with the tested compounds used in their IC_50_ concentrations, then cells were harvested, stained with Annexin V-FITC and PI, and analyzed using flow cytometry. Data are expressed as % of cells in the early stage of apoptosis, and as % of cells in the late stage of apoptosis or necrosis. Data are expressed as the mean ± SD. *** *p* ≤ 0.001, ** *p* ≤ 0.01, as compared to the control.

**Figure 3:**
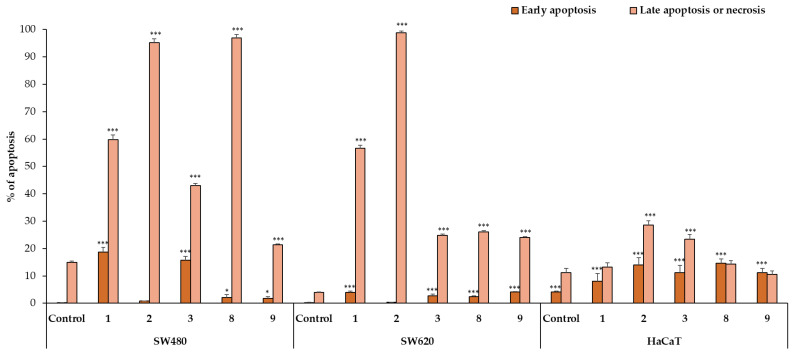
The effect of compounds **1**–**3**, **8**, and **9** on early and late apoptosis in PC3 and K-562 cells. Cells were incubated for 72 h with the tested compounds used in their IC_50_ concentrations, then cells were harvested, stained with Annexin V-FITC and PI, and analyzed using flow cytometry. Data are expressed as % of cells in the early stage of apoptosis, and as % of cells in the late stage of apoptosis. Data are expressed as the means ± SD. *** *p* ≤ 0.001, * *p* ≤ 0.05, as compared to the control.

**Figure 4:**
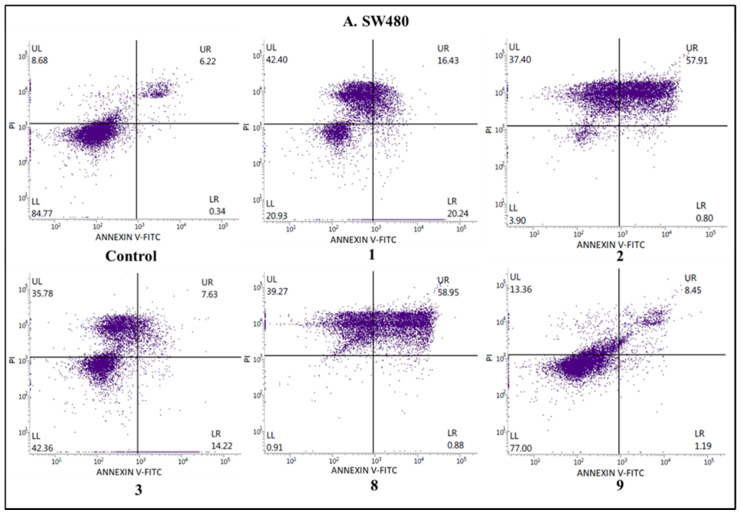

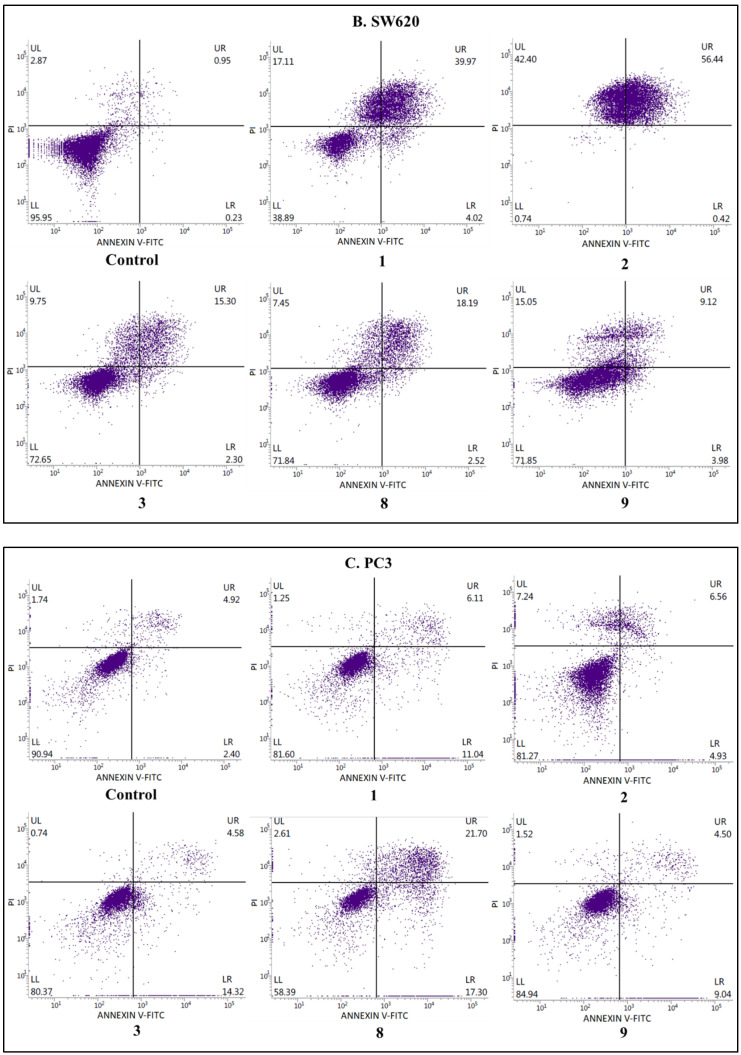

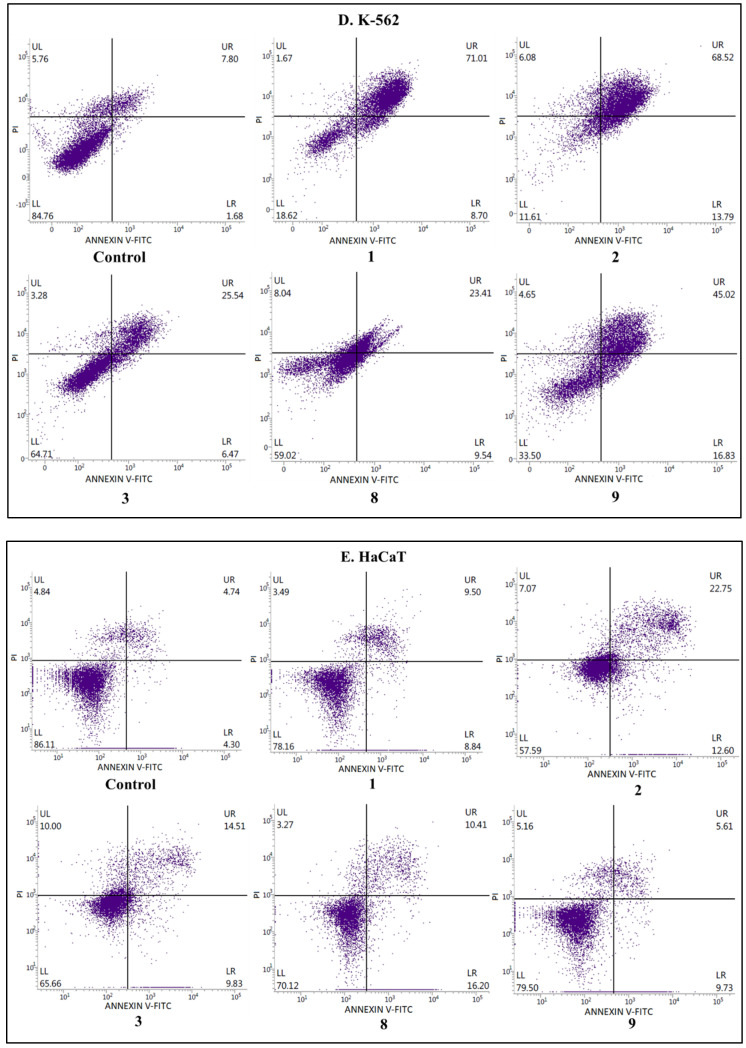
The effects of compounds **1**, **2**, **3**, **8**, and **9** on early and late apoptosis or necrosis in (**A**) SW480, (**B**) SW620, (**C**) PC3, (**D**) K-562, and (**E**) HaCaT cells detected with Annexin V-FITC/PI by flow cytometry. Cells were incubated for 72 h with the tested compounds. Dot plot diagrams show representative experiments. The lower left (LL) quadrant represents viable cells and the lower right (LR) quadrant early apoptotic cells. The upper right (UR) quadrant contains late-stage apoptotic cells, and the upper left (UL) quadrant necrotic cells.

To estimate the mechanism of anticancer activity of the selected compounds **1**–**3**, **8**, and **9**, their effect on both early and late apoptosis was evaluated by flow cytometry analysis. As shown in Figure 2, Figure 3 and Figure 4, the studied derivatives applied in their IC_50_ concentrations induced considerably late apoptosis or necrosis in cancerous cells compared to controls. The apoptosis-activating effect was the strongest in the SW480, SW620, and K-562 cell lines, and the most noticeable activity was observed for the thiourea **2**, followed by its analogs **1** and **8**. Dichlorophenyl derivative **2** and fluorinated thiourea **8** showed a very high percentage of SW480 cells in late apoptosis (95% ± 1.5% and 97% ± 1.2%, respectively). Other tested substances also significantly influenced primary colon cancer cells, causing 24% ± 0.4% (compound **9**) to 60% ± 1.8% (compound **1**) of SW480 cells to be in late apoptosis. The derivative **2** was similarly the most potent activator of apoptosis in metastatic SW620 cells (99% ± 0.5%), in comparison with the thiourea **1** (57% ± 1.2%). The pro-apoptotic impact of derivatives **3**, **8**, and **9** in these cells was not so spectacular and did not exceed 26%. On the other hand, thiourea-derived compounds **1** and **2** considerably affected the level of late apoptosis in leukemia K-562 cells, and gave comparable results (74% ± 3.8% and 73% ± 2.0%, respectively). Additionally, visible late apoptosis-inducing properties in these cell lines were denoted for the compound **9** (47% ± 3.0%), which was twice as strong towards the colon tumor cells mentioned above. The apoptotic properties of analogs **3** and **8** in K-562 cells varied from 30% ± 1.6% to 33% ± 2.3%. In contrary, treatment of PC3 cells with thiourea derivatives did not increase their apoptosis, except for the compound **8** (25% ± 1.5%). The analysis performed in HaCaT cells, incubated with the studied substances **1**, **8**, and **9**, revealed the low level of cells in late apoptosis, counted from 11% to 14%. Whereas the apoptosis-generating influence of derivatives **2** and **3** on normal keratinocytes was higher (29% and 23%), their selectivity measured by MTT methods is favorable. The obtained results are in agreement with the IC_50_ values assigned to pathological cancer cell lines.

#### 2.2.4. Inhibition of IL-6 Release

Interleukin-6 (IL-6) is a cytokine that stimulates the inflammatory and auto-immune processes in many diseases, including pancreatic, prostate, and colon cancers. As its level is higher in advanced and metastatic cancer, IL-6 is also involved in tumor development and progression [37].

Because of the low secretion of IL-6 by leukemic cells compared to solid tumors, and in addition to the lack of IL-6 receptors in K-562 cells [38], studies of the effect of derivatives **1**–**3**, **8**, and **9** on the inhibition of IL-6 release were carried out on primary and secondary solid tumor cells (SW480, SW620, and PC3). The results are given in Table 3 and Figure S2. A remarkable inhibition of the IL-6 level was denoted for both colon cancer cell lines. Compound **2**, identified as the strongest inhibitor, reduced the interleukin level by 54% (in SW480 cells) and 63% (SW620 cells). A significant effect was also observed in these cells for its structural isomer **3** (39% and 23% of inhibition, respectively). Additionally, compound **3** considerably affected the IL-6 level produced by PC3 cells, and reduced it by 33%. Similarly, the treatment with derivative **1** was effective for both colon cancer cells. In its presence, IL-6 release decreased by 28–36%, as compared to controls. For monosubstituted derivatives **8** and **9**, the observed IL-6-reducing influence was weaker. They both inhibited IL-6 secretion in SW480 and SW620 cell lines by 25–32%. In contrast to compound **9**, thiourea **8** additionally diminished the cytokine level in PC3 cells by 26%.

## 3. Materials and Methods

### 3.1. Chemistry

#### 3.1.1. General Procedure

(Trifluoromethyl)aniline was supplied by Alfa Aesar (Stock No. A15910). Isothiocyanates were purchased from Alfa Aesar or Sigma Aldrich. Acetonitrile, chloroform, and methanol were supplied by POCh (Polskie Odczynniki Chemiczne). All chemicals were of analytical grade and were used without any further purification. Prior to usage, acetonitrile was kept in crown cap bottles over anhydrous phosphorus pentoxide (Carl Roth, Karlsruhe, Germany). The NMR spectra were recorded on a Bruker AVANCE DMX400 spectrometer, operating at 300 (^1^H NMR) and 75.5 MHz (^13^C NMR). The spectra were measured in DMSO and are given as δ values (in ppm) relative to TMS. Mass spectral ESI measurements were carried out on an LCT Micromass TOF HiRes apparatus. Flash chromatography was performed on Merck silica gel 60 (200–400 mesh) using chloroform:methanol mixture. Analytical TLC was carried out on silica gel F254 (Merck, Darmstadt, Germany) plates (0.25 mm thickness).

##### General Procedure for the Preparation of N-aryl-[3-(trifluoromethyl)phenyl]thiourea Derivatives (**1**–**12**)

A solution of commercially available 3-(trifluoromethyl)aniline (00031 mol, 0.50 g) in anhydrous acetonitrile (5 mL) was treated with appropriate isothiocyanate (0.0031 mol) and the mixture was stirred at room temperature for 24 h. Then, solvent was removed on a rotary evaporator. The residue was purified by column chromatography (chloroform: methanol; 9.5:0.5 vol.) to yield derivatives **1**–**12**.

*1-(3-chloro-4-fluorophenyl)-3-[3-(trifluoromethyl)phenyl]thiourea* (**1**) was synthesized as described previously [39].

*1-(3,4-dichlorophenyl)-3-[3-(trifluoromethyl)phenyl]thiourea* (**2**) was synthesized as described previously [39].

*1-(2,4-dichlorophenyl)-3-[3-(trifluoromethyl)phenyl]thiourea* (**3**) Yield 70%, cream powder, m.p. 155–157 °C. ^1^H NMR (300 MHz, DMSO) δ (ppm): 10.22 (s, 1H, NH), 9.73 (s, 1H, NH), 8.00 (s, 1H, Ar-H), 7.80–7.77 (d, 1H, *J* = 9.0 Hz, Ar-H), 7.71–7.70 (d, 1H, *J* = 3.0 Hz, Ar-H), 7.62–7.55 (m, 2H, Ar-H), 7.50–7.43 (m, 2H, Ar-H). ^13^C NMR (75.5 MHz, DMSO) δ (ppm): 180.62, 140.11, 135.34, 131.49, 131.30, 131.07, 129.62, 129.06 (q, *J_C-F_* = 31.7 Hz), 129.06, 128.43, 127.48, 124.03 (q, *J_C-F_* = 272.6 Hz), 120.97 (q, *J_C-F_* = 3.8 Hz), 119.91 (q, *J_C-F_* = 3.8 Hz). HRMS (ESI) calc. for C_14_H_8_N_2_F_3_SCl_2_ [M − H]^−^: 362.9726, found: 362.9737.

*1-(2,3-dichlorophenyl)-3-[3-(trifluoromethyl)phenyl]thiourea* (**4**) Yield 65%, white powder, m.p. 140–142 °C. ^1^H NMR (300 MHz, DMSO) δ (ppm): 10.24 (s, 1H, NH), 9.83 (s, 1H, NH), 7.99 (s, 1H, Ar-H), 7.80–7.77 (d, 1H, *J* = 9.0 Hz, Ar-H), 7.61–7.53 (m, 3H, Ar-H), 7.50–7.47 (m, 1H, Ar-H), 7.41–7.35 (m, 1H, Ar-H). ^13^C NMR (75.5 MHz, DMSO) δ (ppm): 180.63, 140.13, 138.17, 131.90, 129.62, 129.32, 129.05 (q, *J_C-F_* = 31.7 Hz), 128.63, 128.35, 127.88, 127.47, 124.03 (q, *J_C-F_* = 272.6 Hz), 120.98 (q, *J_C-F_* = 3.8 Hz), 119.93 (q, *J_C-F_* = 4.5 Hz). HRMS (ESI) calc. for C_14_H_8_N_2_F_3_SCl_2_ [M − H]^−^: 362.9726, found: 362.9737.

*1-(3-chloro-2-methylphenyl)-3-[3-(trifluoromethyl)phenyl]thiourea* (**5**) Yield 65%, white powder, m.p. 163–165 °C. ^1^H NMR (300 MHz, DMSO) δ (ppm): 9.95 (s, 1H, NH), 9.77 (s, 1H, NH), 7.94 (m, 1H, Ar-H), 7.79–7.75 (m, 1H, Ar-H), 7.61–7.44 (m, 2H, Ar-H), 7.41–7.34 (m, 1H, Ar-H), 7.26–7.23 (m, 2H, Ar-H), 2.27 (s, 3H, CH_3_). ^13^C NMR (75.5 MHz, DMSO) δ (ppm): 180.80, 140.43, 139.07, 133.85, 133.50, 129.44, 129.36 (q, *J_C-F_* = 31.7 Hz), 128.73, 127.51, 127.23, 127.10, 124.07 (q, *J_C-F_* = 272.6 Hz), 120.72 (q, *J_C-F_* = 3.8 Hz), 119.98 (q, *J_C-F_* = 4.5 Hz), 15.27. HRMS (ESI) calc. for C_15_H_11_N_2_F_3_SCl [M − H]^−^: 343.0315, found: 343.0284.

*1-(5-chloro-2-methylphenyl)-3-[3-(trifluoromethyl)phenyl]thiourea* (**6**) Yield 63%, cream powder, m.p. 146–148 °C. ^1^H NMR (300 MHz, DMSO) δ (ppm): 10.03 (s, 1H, NH), 9.64 (s, 1H, NH), 7.96–7.93 (m, 1H, Ar-H), 7.79–7.74 (m, 1H, Ar-H), 7.59–7.54 (t, 1H, *J* = 7.5 Hz, Ar-H), 7.50–7.45 (m, 1H, Ar-H), 7.39–7.38 (d, 1H, *J* = 3.0 Hz, Ar-H), 7.31–7.28 (m, 1H, Ar-H), 7.26–7.20 (m, 1H, Ar-H), 2.23 (s, 3H, CH_3_). ^13^C NMR (75.5 MHz, DMSO) δ (ppm): 180.55, 140.34, 138.83, 133.84, 131.85, 129.85, 129.51, 128.99 (q, *J_C-F_* = 31.7 Hz), 127.52, 126.43, 125.86, 124.10 (q, *J_C-F_* = 271.8 Hz), 120.75 (q, *J_C-F_* = 4.5 Hz), 119.95 (q, *J_C-F_* = 3.8 Hz), 17.29. HRMS (ESI) calc. for C_15_H_11_N_2_F_3_SCl [M − H]−: 343.0315, found: 343.0284.

*1-[2-(trifluoromethyl)phenyl]-3-[3-(trifluoromethyl)phenyl]thiourea* (**7**) Yield 72%, white powder, m.p. 173–175 °C. ^1^H NMR (300 MHz, DMSO) δ (ppm): 10.18 (s, 1H, NH), 9.60 (s, 1H, NH), 8.05 (s, 1H, Ar-H), 7.78–7.68 (m, 3H, Ar-H), 7.60–7.47 (m, 4H, Ar-H). ^13^C NMR (75.5 MHz, DMSO) δ (ppm): 181.51, 140.17, 136.79, 132.81, 132.33, 129.62, 129.05 (q, *J_C-F_* = 31.7 Hz), 128.42, 127.52, 127.26, 126.25 (q, *J_C-F_* = 4.5 Hz), 125.85, 125.38, 120.91 (q, *J_C-F_* = 3.8 Hz), 119.74 (q, *J_C-F_* = 3.8 Hz). HRMS (ESI) calc. for C_15_H_9_N_2_F_6_S [M − H]^−^: 363.0368, found: 363.0391.

*1-[3-(trifluoromethyl)phenyl]-3-[4-(trifluoromethyl)phenyl]thiourea* (**8**) Yield 76%, white powder, m.p. 134–136 °C. ^1^H NMR (300 MHz, DMSO) δ (ppm): 10.36–10.34 (m, 1H, NH), 10.26–10.23 (m, 1H, NH), 7.96–7.93 (m, 1H, Ar-H), 7.79–7.68 (m, 5H, Ar-H), 7.61–7.55 (t, 1H, *J* = 9.0 Hz, Ar-H), 7.50–7.47 (m, 1H, Ar-H). ^13^C NMR (75.5 MHz, DMSO) δ (ppm): 179.90, 143.02, 140.14, 129.64, 129.32, 128.48, 127.43, 126.13, 125.73 (q, *J_C-F_* = 3.8 Hz), 124.26, 124.27 (q, *J_C-F_* = 32.5 Hz), 123.06, 122.38, 120.94 (q, *J_C-F_* = 3.8 Hz), 119.91 (q, *J_C-F_* = 3.9 Hz). HRMS (ESI) calc. for C_15_H_9_N_2_F_6_S [M − H]^−^: 363.0368, found: 363.0391.

*1-(4-chlorophenyl)-3-[3-(trifluoromethyl)phenyl]thiourea* (**9**) was synthesized as described previously [2].

*1-(4-cyanophenyl)-3-[3-(trifluoromethyl)-phenyl]thiourea* (**10**) was synthesized as described previously [2].

*1-(4-bromophenyl)-3-[3-(trifluoromethyl)phenyl]thiourea* (**11**) Yield 75%, white powder, m.p. 182–184 °C. ^1^H NMR (300 MHz, DMSO) δ (ppm): 10.09–10.08 (d, 2H, *J* = 3.0 Hz, NH), 7.94 (s, 1H, Ar-H), 7.77–7.74 (d, 1H, *J* = 9.0 Hz, Ar-H), 7.59–7.52 (m, 3H, Ar-H), 7.48–7.44 (m, 3H, Ar-H). ^13^C NMR (75.5 MHz, DMSO) δ (ppm): 179.82, 140.31, 138.52, 131.39, 129.52, 128.6 (q, *J_C-F_* = 31.0 Hz), 128.38, 127.36, 125.72, 124.06 (q, *J_C-F_* = 272.6 Hz), 120.73 (q, *J_C-F_* = 3.8 Hz), 119.85 (q, *J_C-F_* = 4.5 Hz), 118.64, 116.81. HRMS (ESI) calc. for C_14_H_9_N_2_F_3_SBr [M − H]^−^: 372.9620, found: 372.9622.

*1-(2-phenylethyl)-3-[3-(trifluoromethyl)-phenyl]thiourea* (**12**) was synthesized as described previously [2].

### 3.2. Biological Studies

#### 3.2.1. Cell Culture

The human primary (SW480), metastatic (SW620) colon cancer, metastatic prostate cancer (PC3), chronic myelogenous leukemia (K-562), and human immortal keratinocyte (HaCaT) cell lines were purchased from the American Type Culture Collection (ATCC, Rockville, MD, USA). The cells were cultured in medium according to protocols (MEM for SW480 and SW620, RPMI 1640 for PC3 and K-562, and DMEM for HaCaT cells), supplemented with 10% FBS, penicillin (100 U/mL) and streptomycin (100 μg/mL), and cultured in a 37 °C/5% CO_2_ humidified incubator. The cells were cultured until appropriate confluence was achieved (80–90%). Next, they were harvested by treatment with 0.25% trypsin (Gibco Life Technologies, Waltham, MA, USA) excluding the non-adherent K-562 cell line and used for studies.

#### 3.2.2. MTT Assay

To determine the IC_50_ of the thiourea compounds **1**–**12**, cells were seeded in 96-well plates (1 × 10^4^ cells per well) and treated for 72 h with different concentrations of compounds. Cells without the studied compounds in medium were used as a control.

The cell viability was assessed by determination of MTT salt [3-(4,5-dimethylthiazol-2-yl)-2,5-diphenyltetrazoliumbromide] conversion by mitochondrial dehydrogenase. The MTT assay was performed as previously described [37]. Alternatively, non-adherent leukemic cells were centrifuged in a microplate centrifuge (400× *g*, 5 min) during the collection stages. Experiments were repeated three times. Cell viability was presented as a percent of MTT reduction in the treated cells versus the control cells. The number of viable cells cultured without the studied compounds was assumed as 100%. A decreased relative MTT level indicated decreased cell viability. Thiourea compounds with the highest cytotoxic potential assessed by MTT determination (with the lowest IC_50_) were chosen for subsequent assessments of cytotoxicity mechanisms.

#### 3.2.3. Trypan Blue Assay

Cells (1 × 10^5^ cells per well) were seeded in 12-well plates and after 72 h of incubation with IC_50_ concentrations of the studied compounds **1**–**3**, **8**, and **9**, they were washed twice with PBS (phosphate-buffered saline) and harvested. The live cell count was assessed by the trypan blue exclusion dye assay using an automated cell counter (CountessTM Invitrogen, Waltham, MA, USA). Untreated cells were used as the control.

#### 3.2.4. Annexin V Binding Assay

The cells were cultured and harvested under the conditions described in Section 3.2.1. Then, they were seeded in six-well plates (2 × 10^5^ cells per well), and treated with the selected thioureas **1**–**3**, **8**, and **9** at their IC_50_ concentration for 72 h. The effect of these compounds on the process of early and late apoptosis and necrosis was determined as described previously [37] by dual staining with Annexin V:FITC and propidium iodide, according to the manufacturer’s protocol (Becton Dickinson). The cells that were Annexin V:FITC positive and PI negative were identified as early apoptotic, and Annexin V:FITC and PI positive as late apoptotic or necrotic.

#### 3.2.5. Il-6 Level Assay

The IL-6 concentration in all studied cancer cells and normal HaCaT cell lines was measured by an ELISA kit (Diaclon SAS Besancon CEDEX, Besançon, France). Cells were seeded in 12-well plates (1 × 10^5^ cells per well) and treated with the IC_50_ concentration of the studied compounds **1**–**3**, **8**, and **9** for 72 h. IL-6 in cell culture supernatant was measured using the enzyme-linked immunosorbent assay in accordance with the manufacturer’s protocol.

#### 3.2.6. Statistical Analyses

Statistical analyses were performed using GraphPad Prism 9 software (GraphPad Software). The results were expressed as mean ± SD from at least three independent experiments. The statistical significance of differences between means was established by ANOVA with Dunnett’s multiple comparison post hoc test. P values below 0.05 were considered statistically significant.

## 4. Conclusions

In summary, we herein report a high-throughput synthesis of 3-(trifluoromethyl)aniline with various isothiocyanates. The effect of all the synthesized compounds’ inhibition of the growth of human tumor cell lines, such as SW480 (primary colon cancer), SW620 (metastatic colon cancer), PC3 (metastatic prostate cancer), and K-562 (chronic myelogenous leukemia), was evaluated. Dihalogenophenyl (**1**–**4**) and *para*-substituted thioureas (**8**, **9**) were highly cytotoxic against the mentioned pathological cell cultures (IC_50_ ≤ 10 µM), with selectivity over normal HaCaT cells. Compounds **2**, **3**, and **9** were more effective and selective than the reference cisplatin. The mechanisms of the in vitro cytotoxic activity of the most bioactive compounds **1**–**3**, **8**, and **9** were studied. All of them were cytostatic and reduced the cancer cells’ number, being safe for normal keratinocytes. Cytotoxic thioureas **1**, **2**, and **9** considerably diminished the viability of both SW480 and SW620 cells. Derivatives **1**, **2**, and **8** exerted the strongest apoptosis-activating effect in SW480, SW620, and K-562 cell lines. Among them, compound **2** showed the highest percentage of colon cancer cells in late apoptosis. The tested derivatives revealed anti-IL-6 activity and significantly decreased the levels of the proinflammatory cytokine produced by both colon carcinoma cells.

The structural modifications of the thiourea terminal moieties indicated the dihalogenophenyl derivative **2**, followed by its isomer **3** and *para*-substituted analog **8**, as the most effective in cancer treatment. This work constitutes an evaluation of the potential and mechanisms of cytotoxic action of thiourea-derived compounds, which will be developed.

## Figures and Tables

**Table 1:** Cytotoxic activity (IC_50_, µM) of hte studied compounds estimated by the MTT assay ^a^.

Compound		Cancer Cells								Normal Cells
		SW480 ^d^		SW620 ^e^		PC3 ^f^		K562 ^g^		HaCaT ^h^
	R	IC_50_^b^	SI ^c^	IC_50_	SI	IC_50_	SI	IC50	SI	IC_50_
**1**	3-Cl,4-F-Ph	12.7 ± 1.53	3.4	9.4 ± 1.85	4.6	53.6 ± 2.94	0.8	6.8 ± 1.57	6.4	43.6 ± 4.22
**2**	3-Cl,4-Cl-Ph	9.0 ± 1.42	2.7	1.5 ± 0.72	16.5	31.7 ± 2.07	0.8	6.3 ± 1.28	3.9	24.7 ± 0.05
**3**	2-Cl,4-Cl-Ph	30.1 ± 3.11	1.7	5.8 ± 0.76	9.0	13.7 ± 7.04	3.8	54.3 ± 1.86	1.0	52.1 ± 0.95
**4**	2-Cl,3-Cl-Ph	22.5 ± 0.10	0.6	14.0 ± 1.73	1.0	10.5 ± 2.52	1.3	35.9 ± 0.07	0.4	13.9 ± 1.70
**5**	2-CH_3_,3-Cl-Ph	7.3 ± 0.89	7.6	23.2 ± 4.07	2.4	51.8 ± 3.19	1.1	52.5 ± 2.14	1.1	55.6 ± 1.74
**6**	2-CH_3_,5-Cl-Ph	15.6 ± 4.10	0.9	22.1 ± 4.36	0.7	65.9 ± 10.50	0.2	40.3 ± 3.88	0.4	14.5 ± 1.08
**7**	2-CF_3_-Ph	>100	0.7	30.1 ± 9.74	2.2	17.1 ± 0.57	3.8	76.5 ± 4.57	0.9	65.7 ± 3.83
**8**	4-CF_3_-Ph	8.9 ± 1.14	4.6	7.6 ± 1.75	5.4	6.9 ± 1.64	6.0	54.8 ± 0.24	0.8	41.3 ± 0.17
**9**	4-Cl-Ph	38.1 ± 4.38	1.9	6.7 ± 1.74	10.7	22.6 ± 1.25	3.2	10.2 ± 0.35	7.4	71.5 ± 3.08
**10**	4-CN-Ph	20.6 ± 3.59	2.4	18.7 ± 5.92	2.7	66.2 ± 4.47	0.8	12.9 ± 4.33	3.9	50.1 ± 4.89
**11**	4-Br-Ph	41.5 ± 4.46	0.4	16.2 ± 4.08	1.1	76.6 ± 4.70	0.2	74.2 ± 6.82	0.2	17.2 ± 0.64
**12**	-CH_2_CH_2_-Ph	25.3 ± 8.53	2.2	38.2 ± 3.10	1.5	26.7 ± 3.95	2.1	23.8 ± 0.45	2.3	55.4 ± 1.08
**Doxorubicin** ^i^	-	0.8 ± 0.10	0.4	0.3 ± 0.08	1.0	0.3 ± 0.12	1.0	0.2 ± 0.10	1.5	0.3 ± 0.11
**Cisplatin** ^j^	-	10.4 ± 0.90	0.6	6.7 ± 1.10	0.9	13.2 ± 2.10	0.5	8.2 ± 4.08	0.8	6.3 ± 0.70

^a^ Data are expressed as mean SD, ^b^ IC_50_ (µM)—the concentration of the compound that corresponds to 50% growth inhibition of the cell line (as compared to the control) after cells were cultured for 72 h with the individual compound. ^c^ The SI (Selectivity Index) was calculated using the formula: SI = IC_50_ for the normal cell line/IC_50_ cancer cell line. ^d^ Human primary colon cancer (SW480), ^e^ Human metastatic colon cancer (SW620), ^f^ Human metastatic prostate cancer (PC3), ^g^ Human chronic myelogenous leukemia (K562), ^h^ Human immortal keratinocyte cell line from adult human skin (HaCaT). ^i,j^ The reference compounds.

**Table 2:** Trypan blue assay. The effect of compounds **1**, **2**, **3**, **8**, and **9** on the live cell number and viability (%) in SW480, SW620, PC3, K-562, and HaCaT cells. Cells were incubated for 72 h with the tested compounds used in their IC_50_ concentrations, then cells were harvested, stained with trypan blue, and analyzed using a cell counter. Data are expressed as the mean ± SD.- “control without compound”, ^a^ Human primary colon cancer (SW480), ^b^ Human metastatic colon cancer (SW620), ^c^ Human metastatic prostate cancer (PC3), ^d^ Human chronic myelogenous leukemia (K562), ^e^ Human immortal keratinocyte cell line from adult human skin (HaCaT).

		Compound	Cell Number × 10^5^	Viability (%)
Cancer cell line	SW480 ^a^	-	3.6 ± 0.20	93 ± 1.35
		1	2.0 ± 0.30	69 ± 1.30
		2	0.3 ± 0.35	45 ± 2.40
		3	1.5 ± 0.10	90 ± 1.30
		8	0.4 ± 0.15	90 ± 2.30
		9	2.5 ± 0.12	72 ± 2.25
	SW620 ^b^	-	5.7 ± 0.10	96 ± 3.30
		1	1.3 ± 0.25	79 ± 1.30
		2	0.4 ± 0.22	42 ± 1.30
		3	2.4 ± 0.20	93 ± 2.33
		8	1.0 ± 0.20	55 ± 1.11
		9	1.6 ± 0.11	84 ± 3.08
	PC3 ^c^	-	3.4 ± 0.25	97 ± 2.30
		1	2.3 ± 0.15	93 ± 1.50
		2	2.4 ± 0.16	93 ± 1.40
		3	1.8 ± 0.18	58 ± 1.20
		8	2.1 ± 0.12	82 ± 1.10
		9	1.2 ± 0.15	73 ± 1.99
	K-562 ^d^	-	4.4 ± 0.15	98 ± 1.30
		1	1.8 ± 0.24	93 ± 2.05
		2	1.0 ± 0.21	55 ± 1.03
		3	3.0 ± 0.27	85 ± 1.20
		8	3.2 ± 0.14	88 ± 1.10
		9	3.5 ± 0.22	90 ± 2.40
Normal cell line	HaCaT ^e^	-	9.2 ± 0.25	98 ± 1.30
		1	6.5 ± 0.15	97 ± 1.33
		2	1.6 ± 0.31	84 ± 1.03
		3	7.8 ± 0.14	96 ± 3.01
		8	4.9 ± 0.28	98 ± 1.02
		9	8.6 ± 0.12	98 ± 1.50

**Table 3:** Effects of compounds **1**–**3**, **8**, and **9** on IL-6 levels, measured by the ELISA test. Data are expressed as the mean ± SD from ^a^ Human primary colon cancer (SW480), ^b^ Human metastatic colon cancer (SW620), ^c^ Human metastatic prostate cancer (PC3), ^d^ Human immortal keratinocyte cell line from adult human skin (HaCaT).

		Compound	IL-6 Concentration (pg/mL)
Cancer cell line	SW480 ^a^	Control	7.8 ± 0.03
		1	5.7 ± 0.01
		2	3.6 ± 0.02
		3	4.8 ± 0.04
		8	5.3 ± 0.01
		9	5.5 ± 0.02
	SW620 ^b^	Control	7.7 ± 0.05
		1	4.9 ± 0.01
		2	2.8 ± 0.03
		3	5.9 ± 0.03
		8	5.8 ± 0.06
		9	5.2 ± 0.03
	PC3 ^c^	Control	10.9 ± 0.07
		1	9.6 ± 0.02
		2	9.1 ± 0.04
		3	7.3 ± 0.06
		8	8.1 ± 0.08
		9	9.9 ± 0.03
Normal cell line	HaCaT ^d^	Control	9.7 ± 0.03
		1	8.7 ± 0.03
		2	9.1 ± 0.03
		3	8.9 ± 0.01
		8	9.1 ± 0.06
		9	9.0 ± 0.01

## Data Availability

Data is contained within the article or supplementary material.

## Supplementary Materials

The following are available online at https://www.mdpi.com/article/10.3390/ph14111097/s1, Figure S1. (A) Trypan blue assay. The effect of compounds **1**, **2**, **3**, **8** and **9** on cell number in SW480, SW620, PC3, K-562 and HaCaT cells. (B) Trypan blue assay. The effect of compounds **1**, **2**, **3**, **8** and **9** on viability in SW480, SW620, PC3, K-562 and HaCaT cells. Figure S2. Effects of compounds **1**–**3**, **8** and **9** on IL-6 levels, measured by ELISA test. ^1^H and ^13^C NMR spectra of synthesized compounds **3**–**8**, **11**. Table S1. High Resolution Mass Spectra (HRMS) of compounds **4**, **6**, **7**, **11**.
