# Supplementary material for: Investigation of the Mechanisms of Cytotoxic Activity of 1,3-Disubstituted Thiourea Derivatives
Source: Pharmaceuticals (Basel). 2021 Oct 28;14(11):1097. doi: 10.3390/ph14111097 (PMC8623398; doi:10.3390/ph14111097)
Supplement: Supplementary file 1 [file pharmaceuticals-14-01097-s001.zip › pharmaceuticals-1419478-supplementary.pdf]

Supplementary material

Investigation of the Mechanisms of Cytotoxic Activity of 1,3-Disubstituted Thiourea Derivatives

Paulina Strzyga-Łach [1], Alicja Chrzanowska [1], Katarzyna Podsadni [1], Anna Bielenica [1,*]

[1] *Chair and Department of Biochemistry, Medical University of Warsaw, 02-097 Warszawa, Poland*

[*]**Corresponding author:** Anna Bielenica, Chair and Department of Biochemistry, Medical University of Warsaw, Banacha 1 Street, 02-097 Warszawa, Poland; Tel: (48)022-572-06-93; Fax: (48)022-572-06-79; e-mail: abielenica@wum.edu.pl

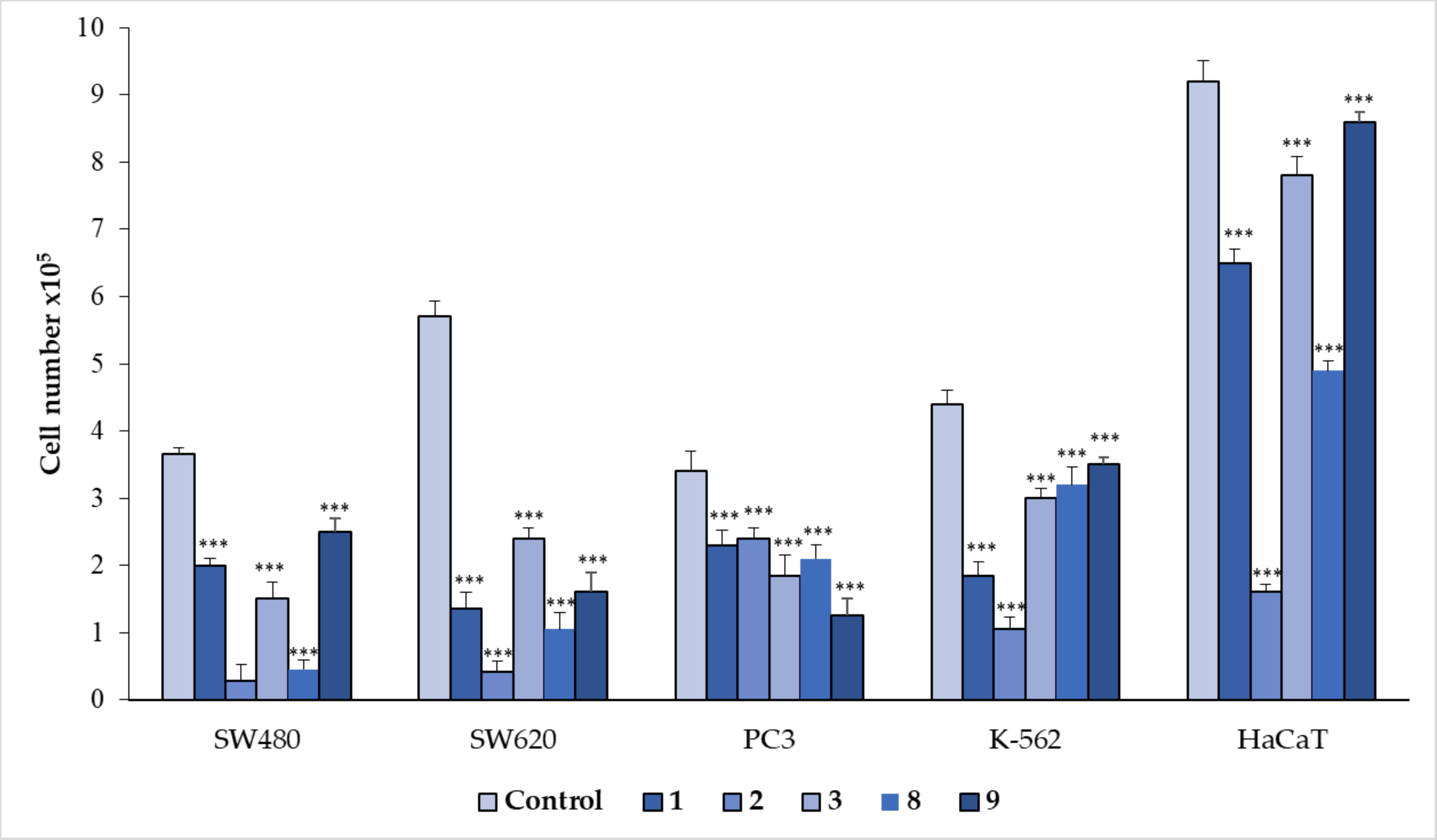

Figure S1A. Trypan blue assay. The effect of compounds **1**, **2**, **3**, **8** and **9** on cell number in SW480, SW620, PC3, K-562 and HaCaT cells. Cells were incubated for 72 h with tested compounds used in their IC_{50} concentrations, then cells were harvested, stained with trypan blue, and analyzed using cell counter. Data are expressed as the mean ± SD. *** p≤0.001, ** p≤0.01, * p≤0.05, as compared to the control.

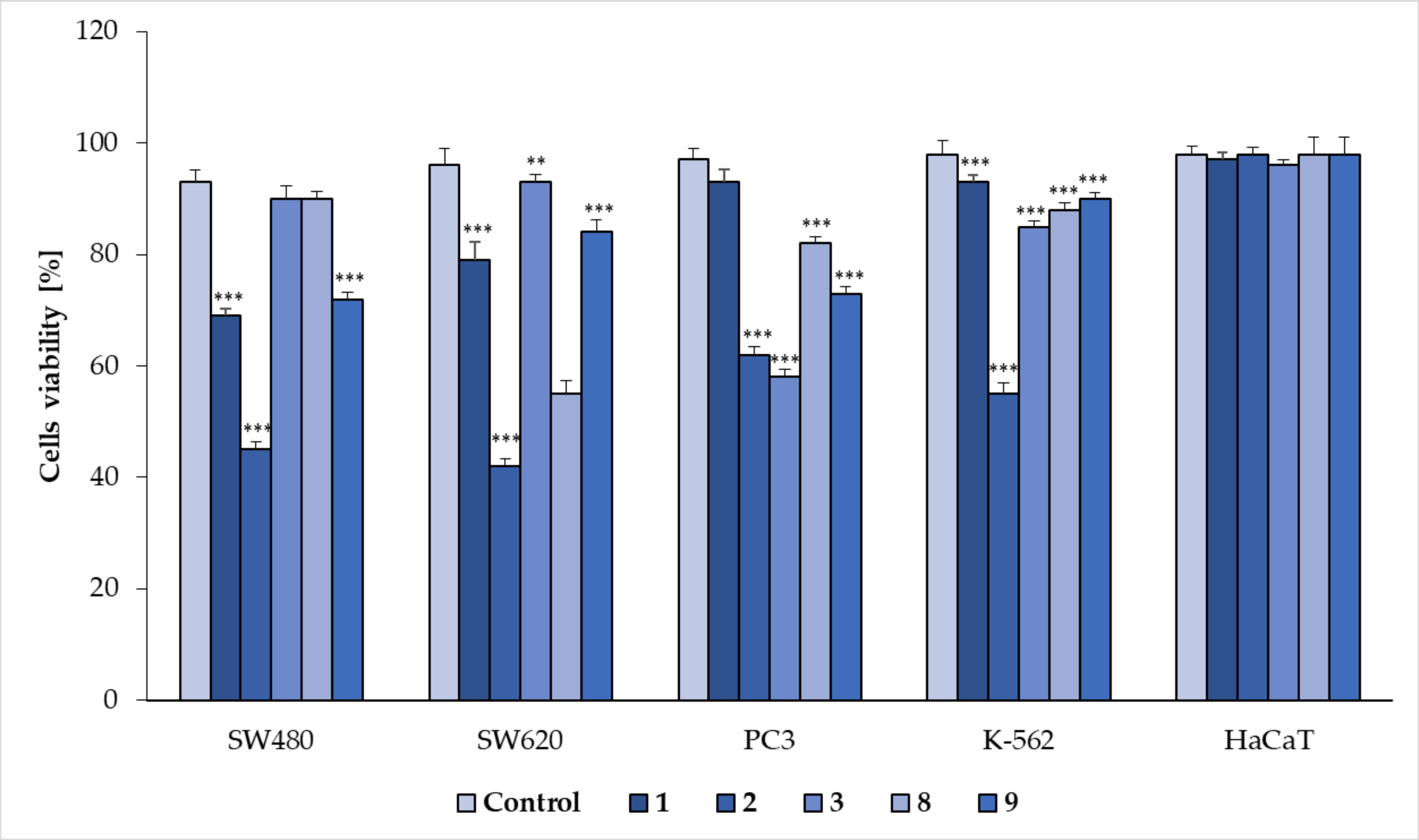

Figure S1B. Trypan blue assay. The effect of compounds **1**, **2**, **3**, **8** and **9** on viability in SW480, SW620, PC3, K-562 and HaCaT cells. Cells were incubated for 72 h with tested compounds used in their IC_{50} concentrations, then cells were harvested, stained with trypan blue, and analyzed using cell counter. Data are expressed as the mean ± SD. *** $p \leq 0.001$, ** $p \leq 0.01$, * $p \leq 0.05$, as compared to the control.

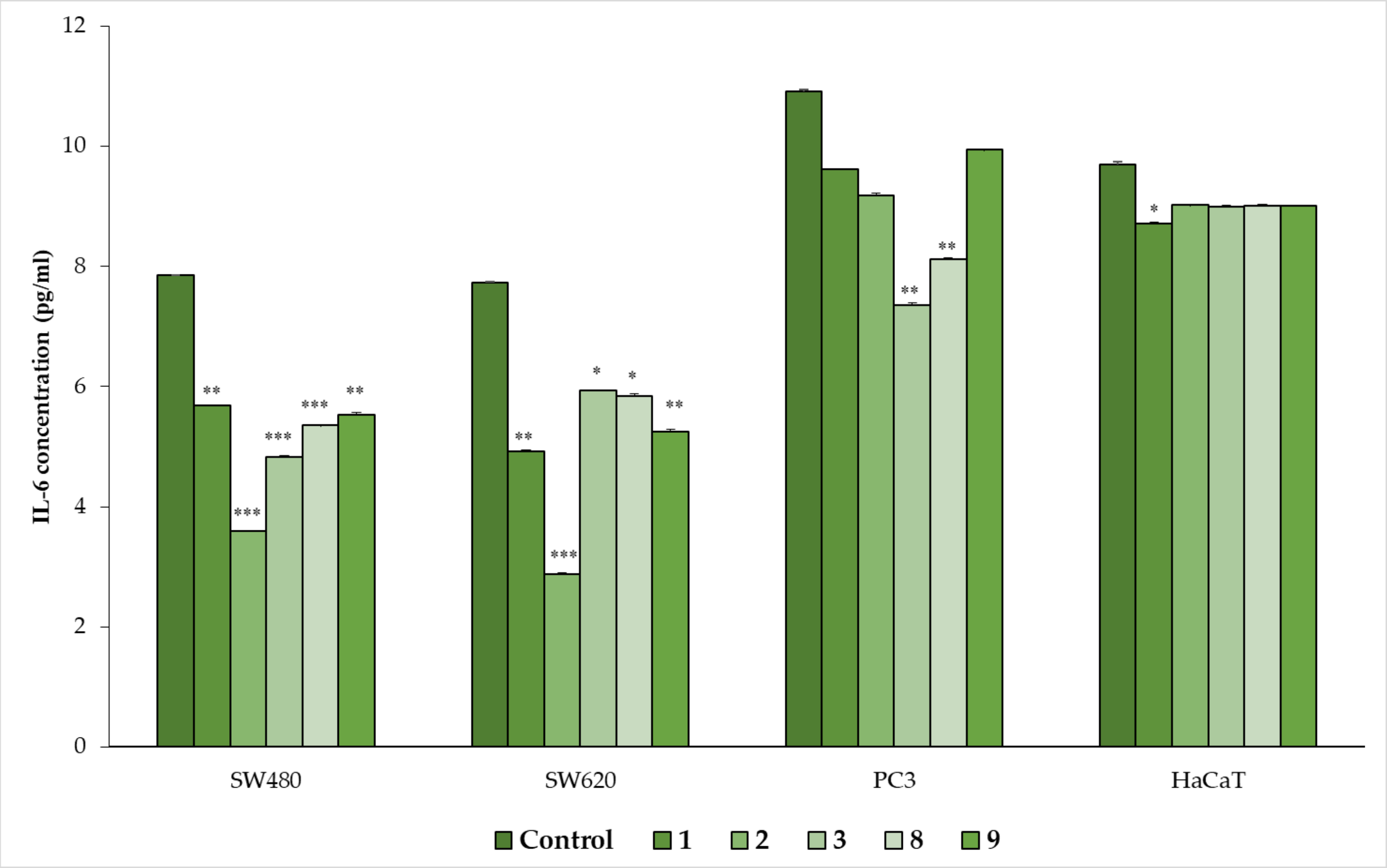

Figure S2. Effects of compounds **1-3, 8** and **9** on IL-6 levels, measured by ELISA test. Data are expressed as the mean ± SD ,*** p≤0.001, ** p≤0.01, * p≤0.05, as compared to the control.

Structural formulas of compounds 3-8, 11.

Compound	Structural formula
3	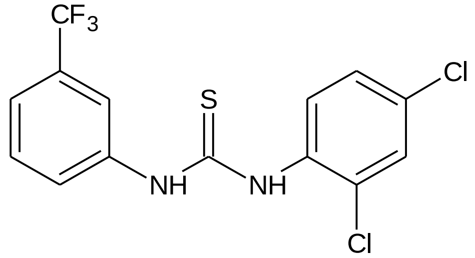
4	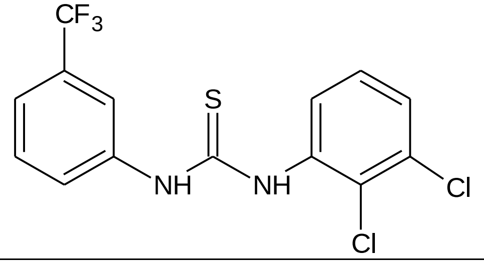
5	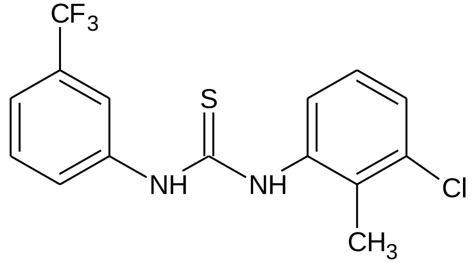

**6**	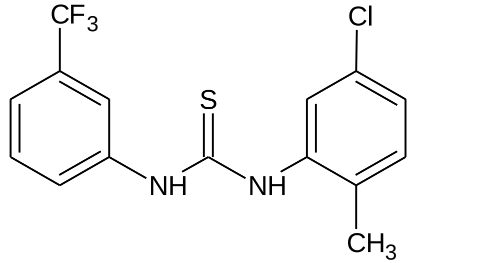CF3<br>S<br>Cl<br>NH<br>NH<br>CH3
**7**	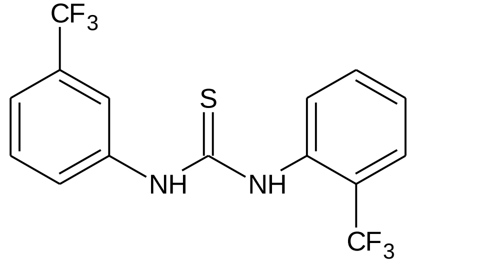CF3<br>S<br>NH<br>NH<br>CF3
**8**	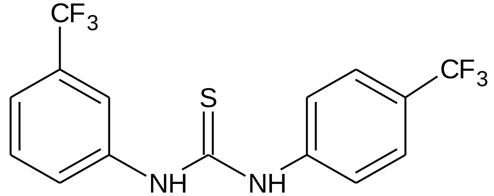CF3<br>S<br>CF3<br>NH<br>NH
**11**	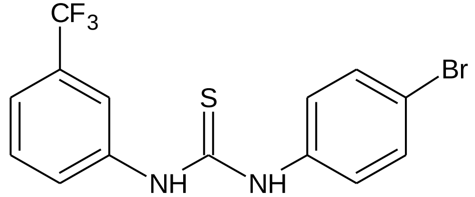CF3<br>S<br>Br<br>NH<br>NH

^{1}H NMR and ^{13}C NMR spectra of compounds 3-8, 11.

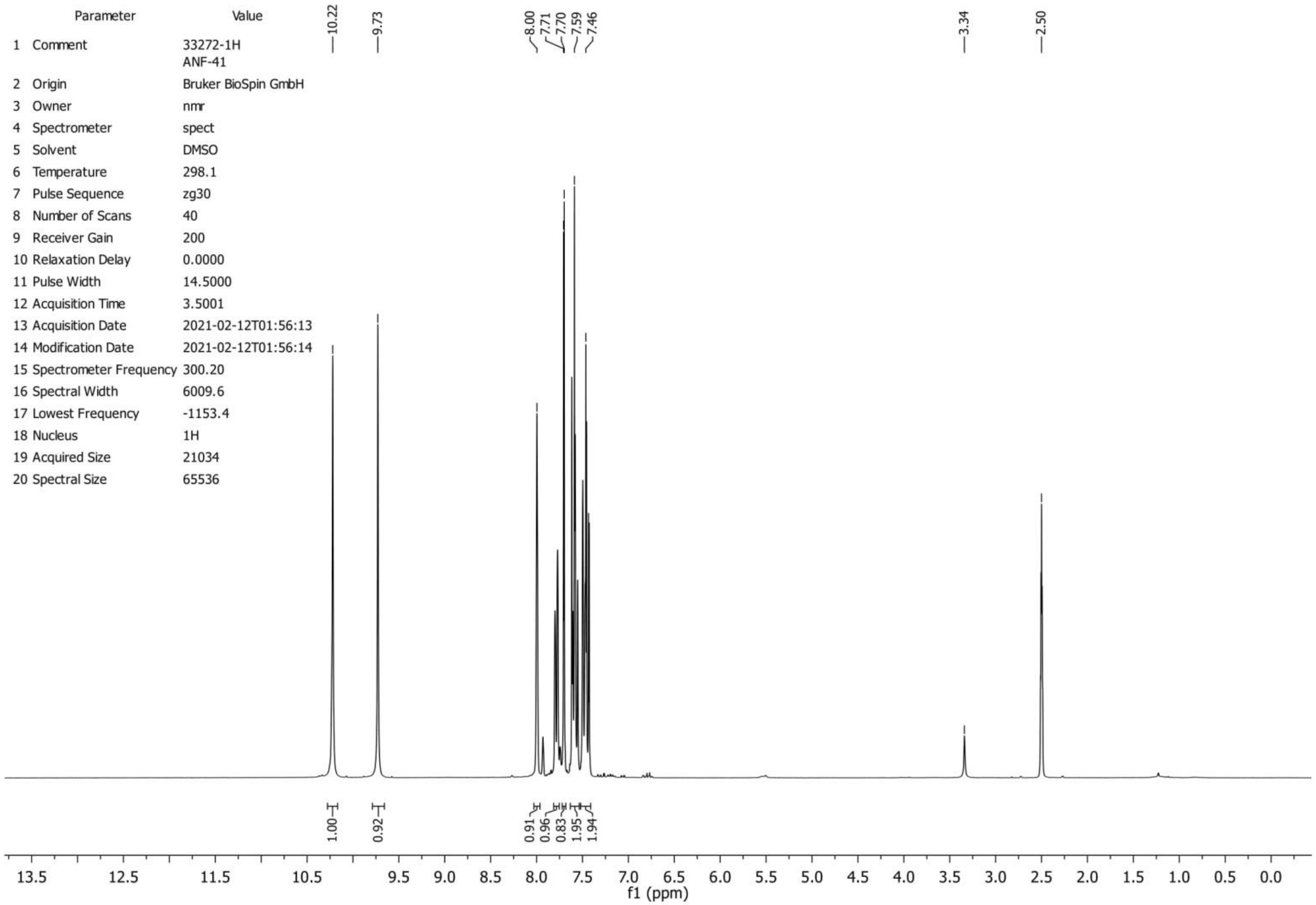

Spectrum 1. ^{1}H NMR of compound **3** (300 MHz, DMSO-d_6).

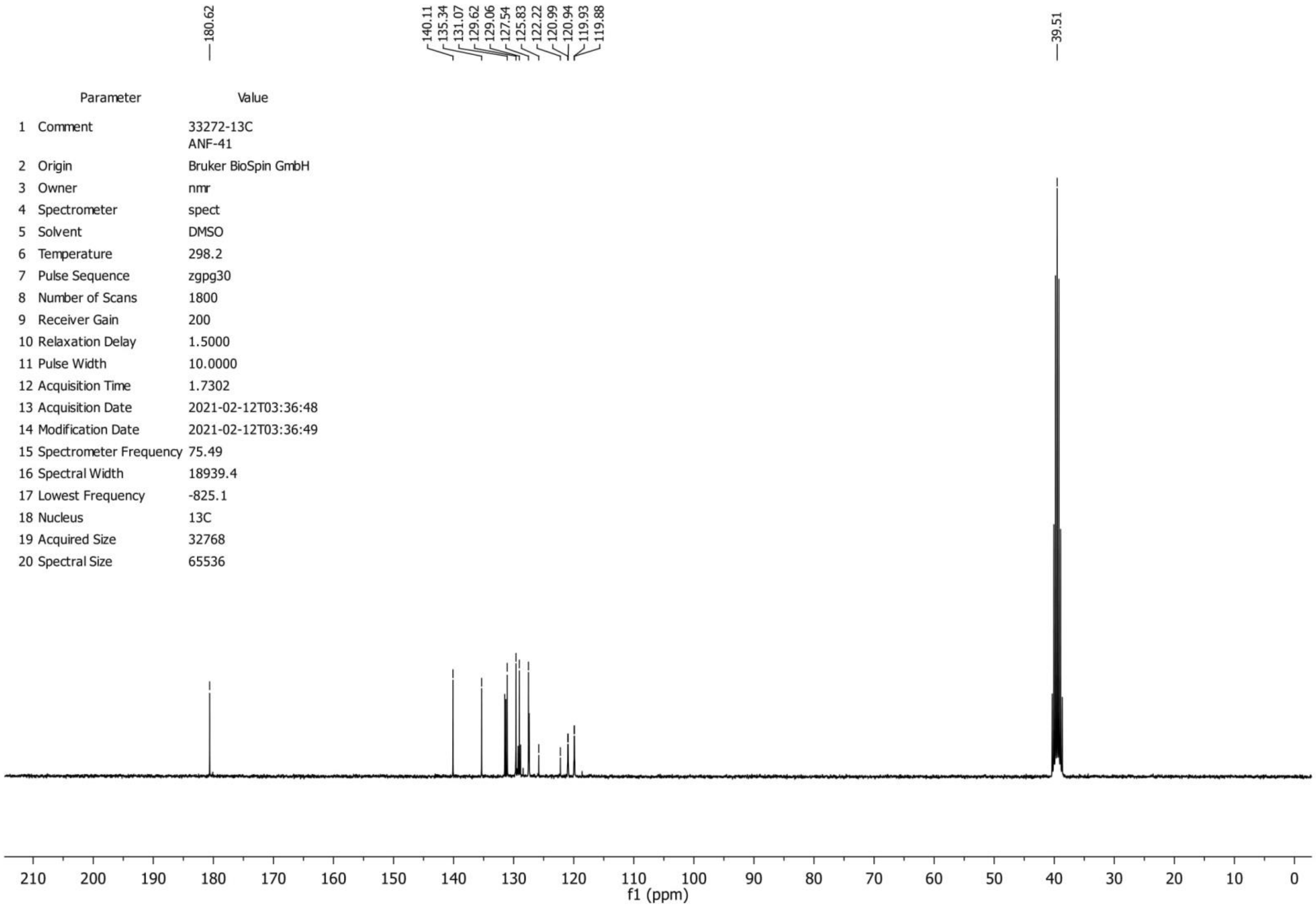

Spectrum 2. ^{13}C NMR of compound **3** (75.5 MHz, DMSO-d_6).

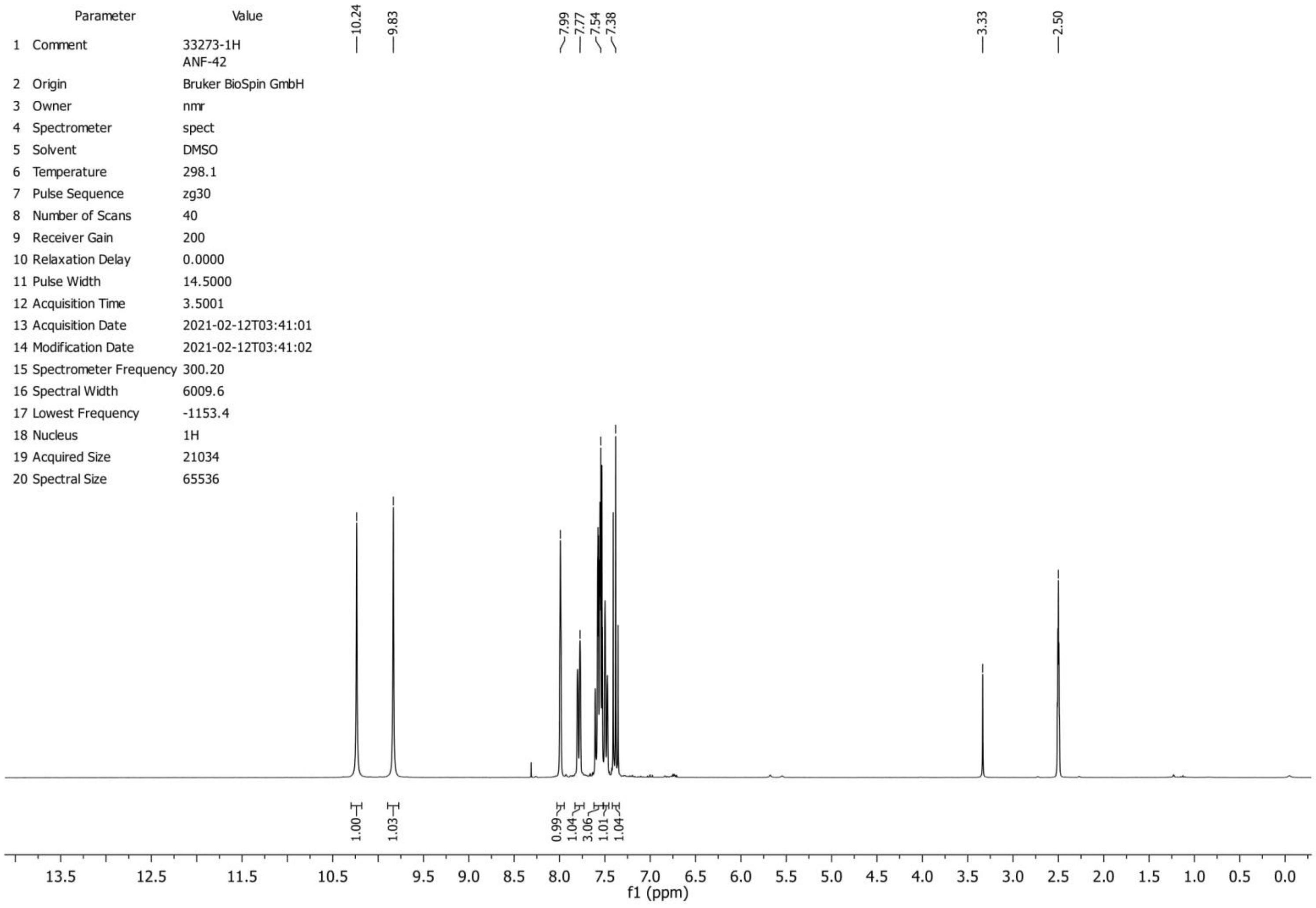

Spectrum 3. ^{1}H NMR of compound **4** (300 MHz, DMSO-d_6).

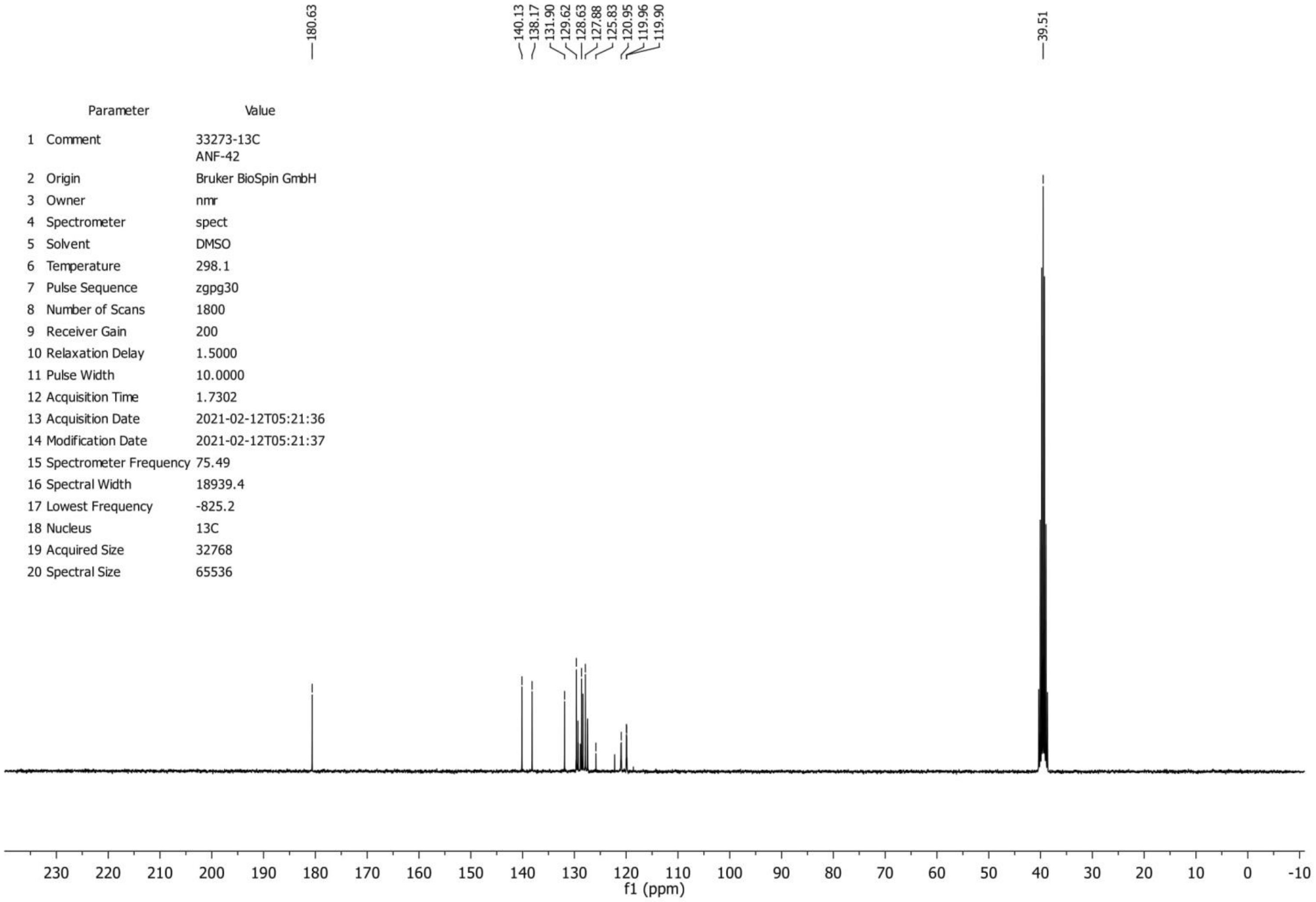

Spectrum 4. ^{13}C NMR of compound **4** (75.5 MHz, DMSO-d_6).

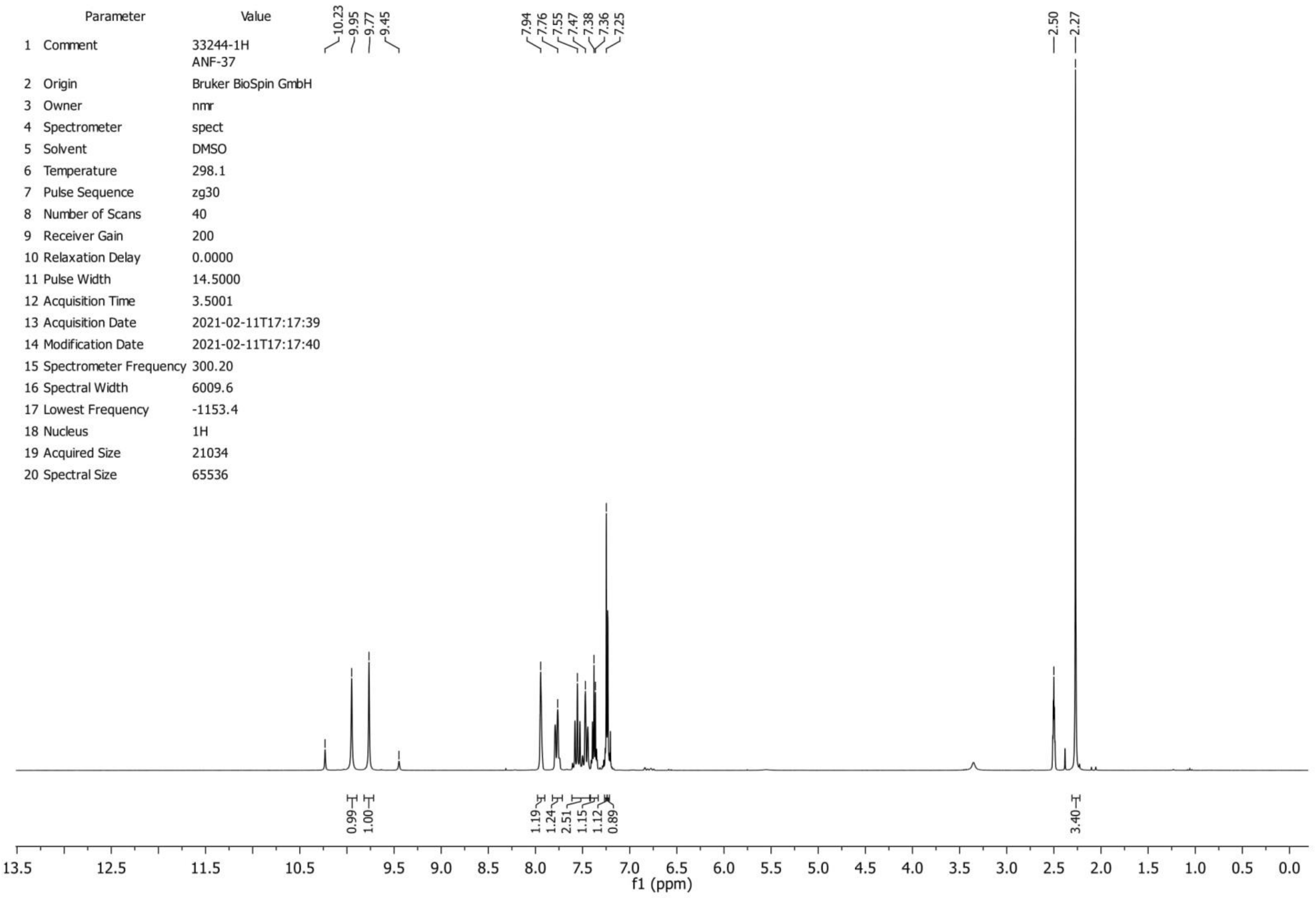

Spectrum 5. ^{1}H NMR of compound **5** (300 MHz, DMSO-d_6).

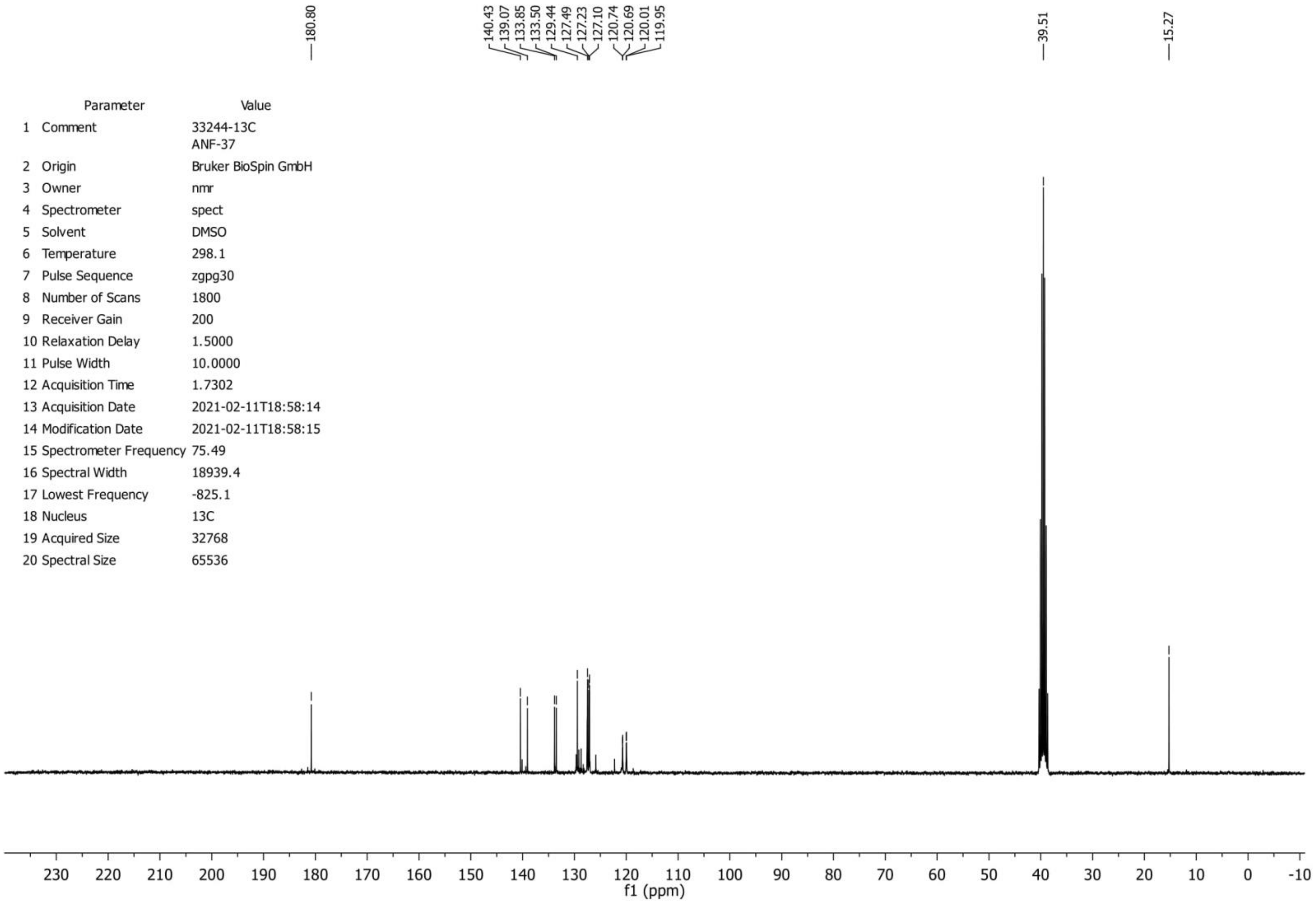

Spectrum 6. ^{13}C NMR of compound **5** (75.5 MHz, DMSO-d_6).

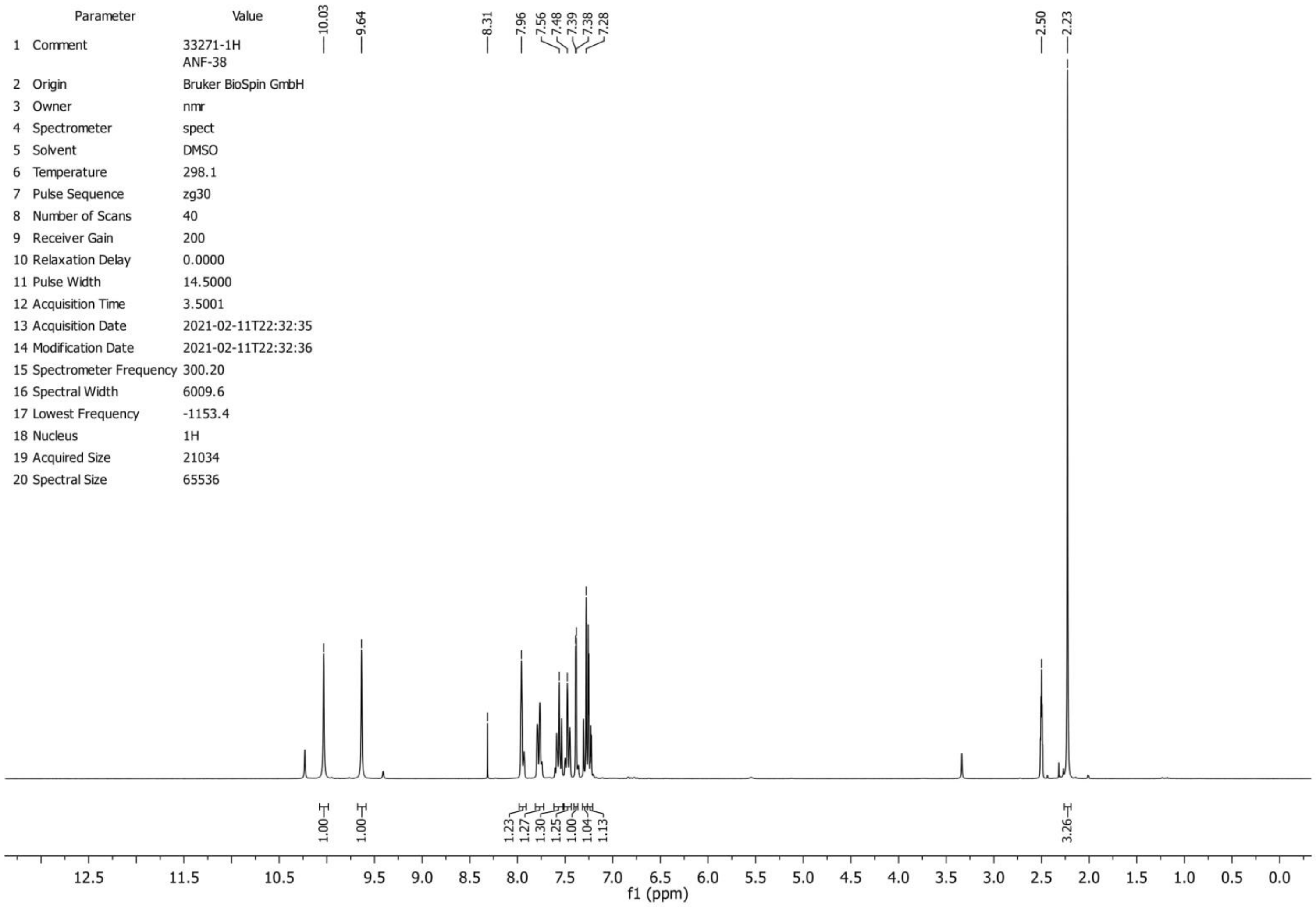

Spectrum 7. ^{1}H NMR of compound **6** (300 MHz, DMSO-d_6).

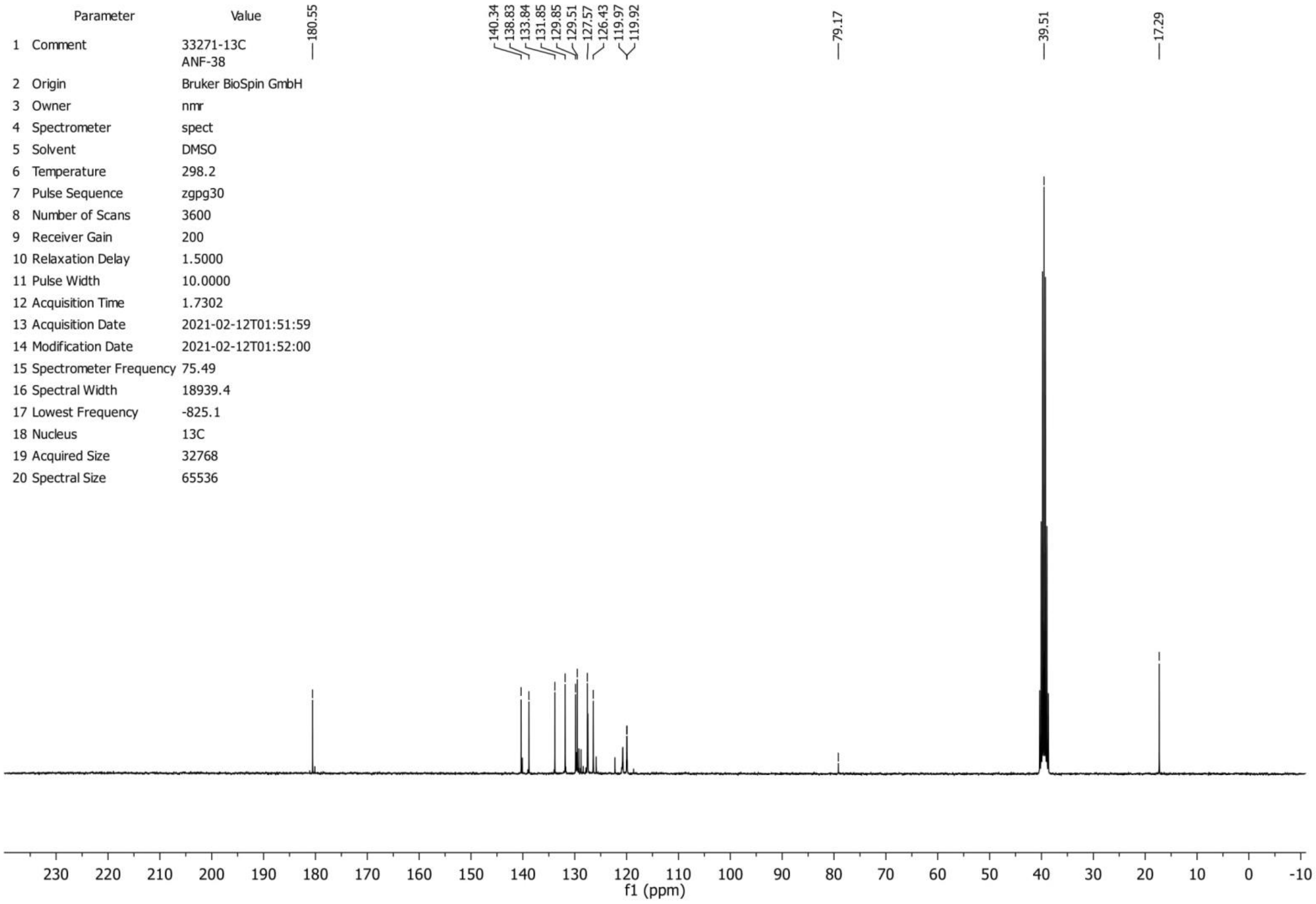

Spectrum 8. ^{13}C NMR of compound **6** (75.5 MHz, DMSO-d_6).

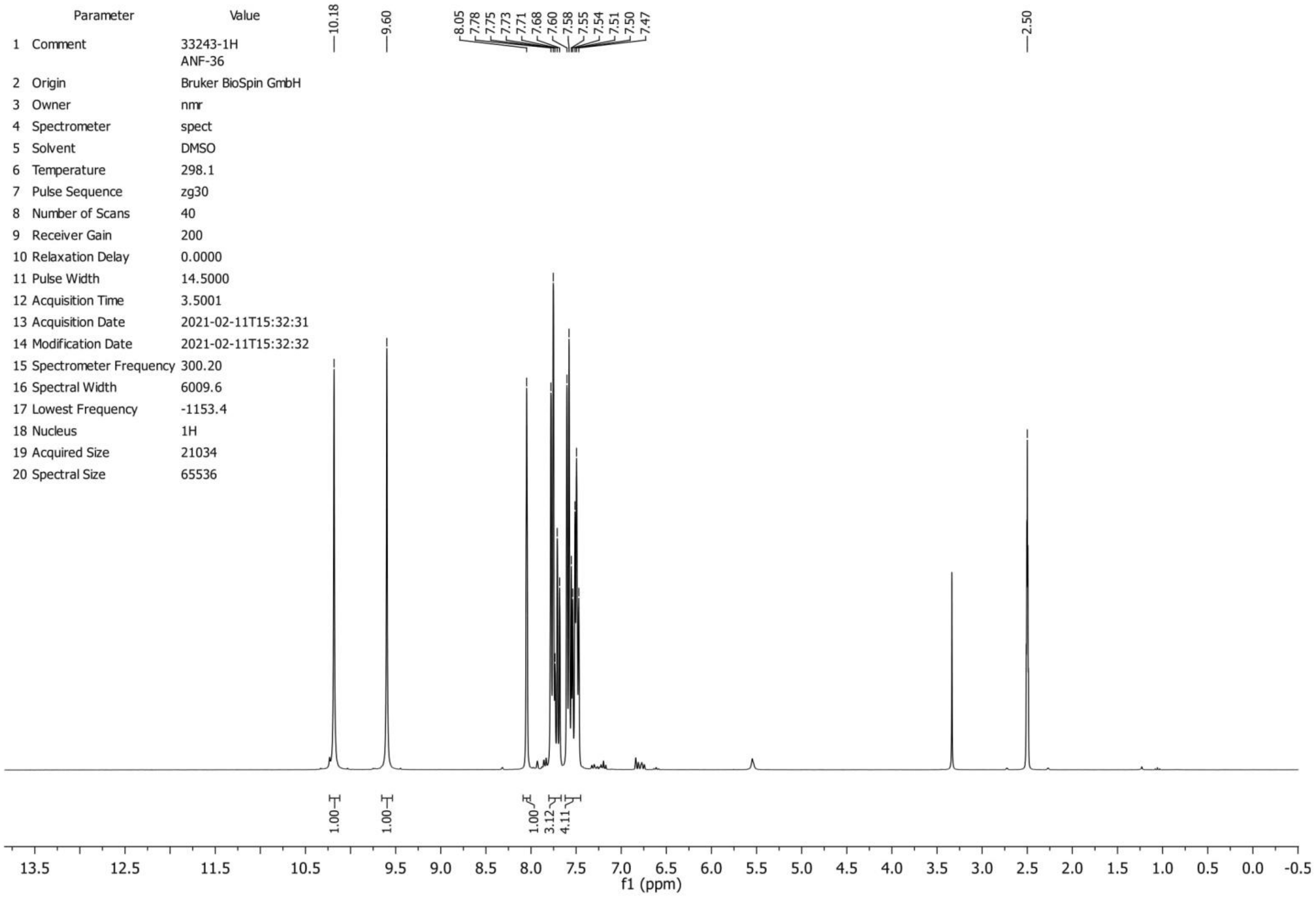

Spectrum 9. ^{1}H NMR of compound **7** (300 MHz, DMSO-d_6).

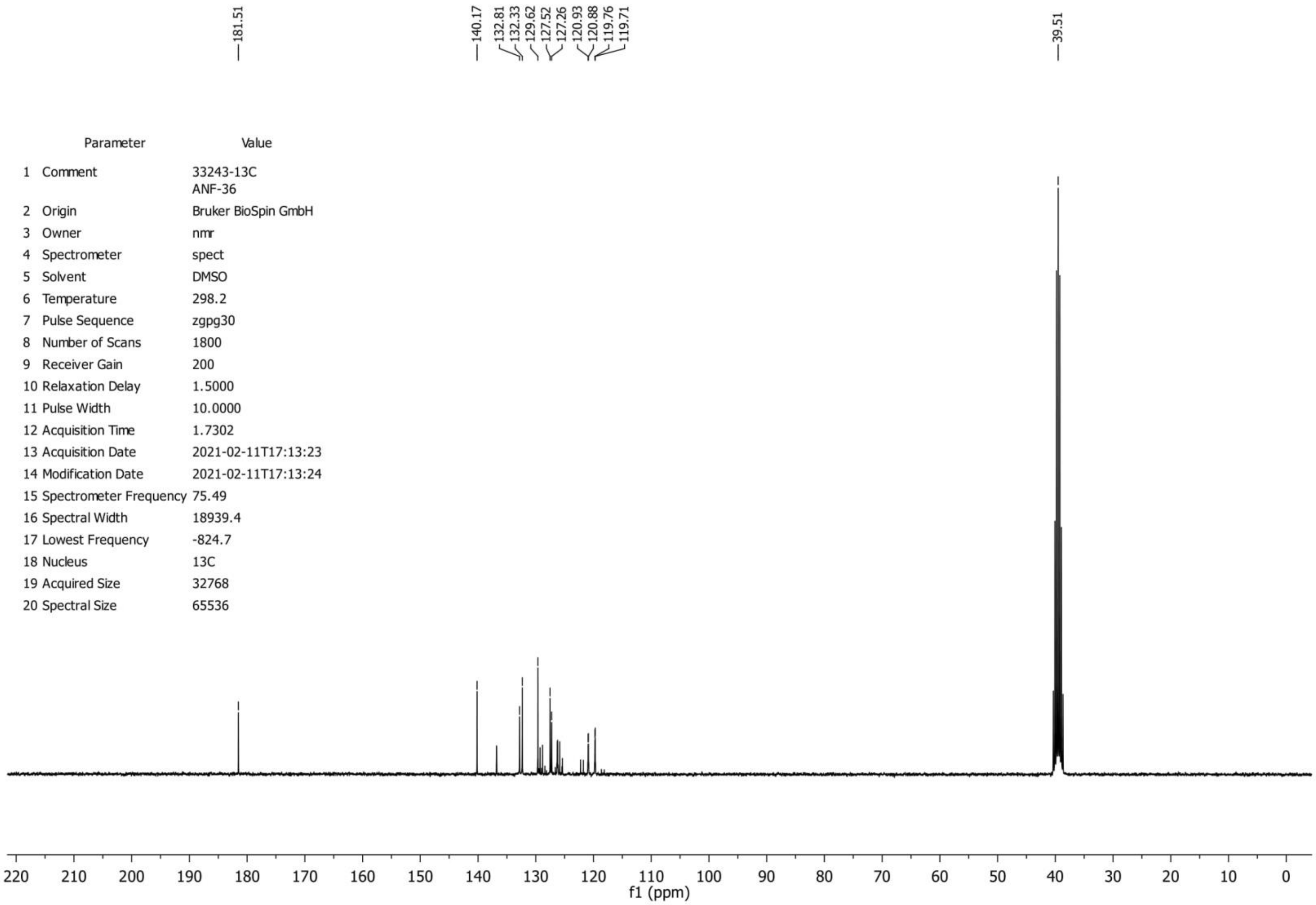

Spectrum 10. ^{13}C NMR of compound **7** (75.5 MHz, DMSO-d_6).

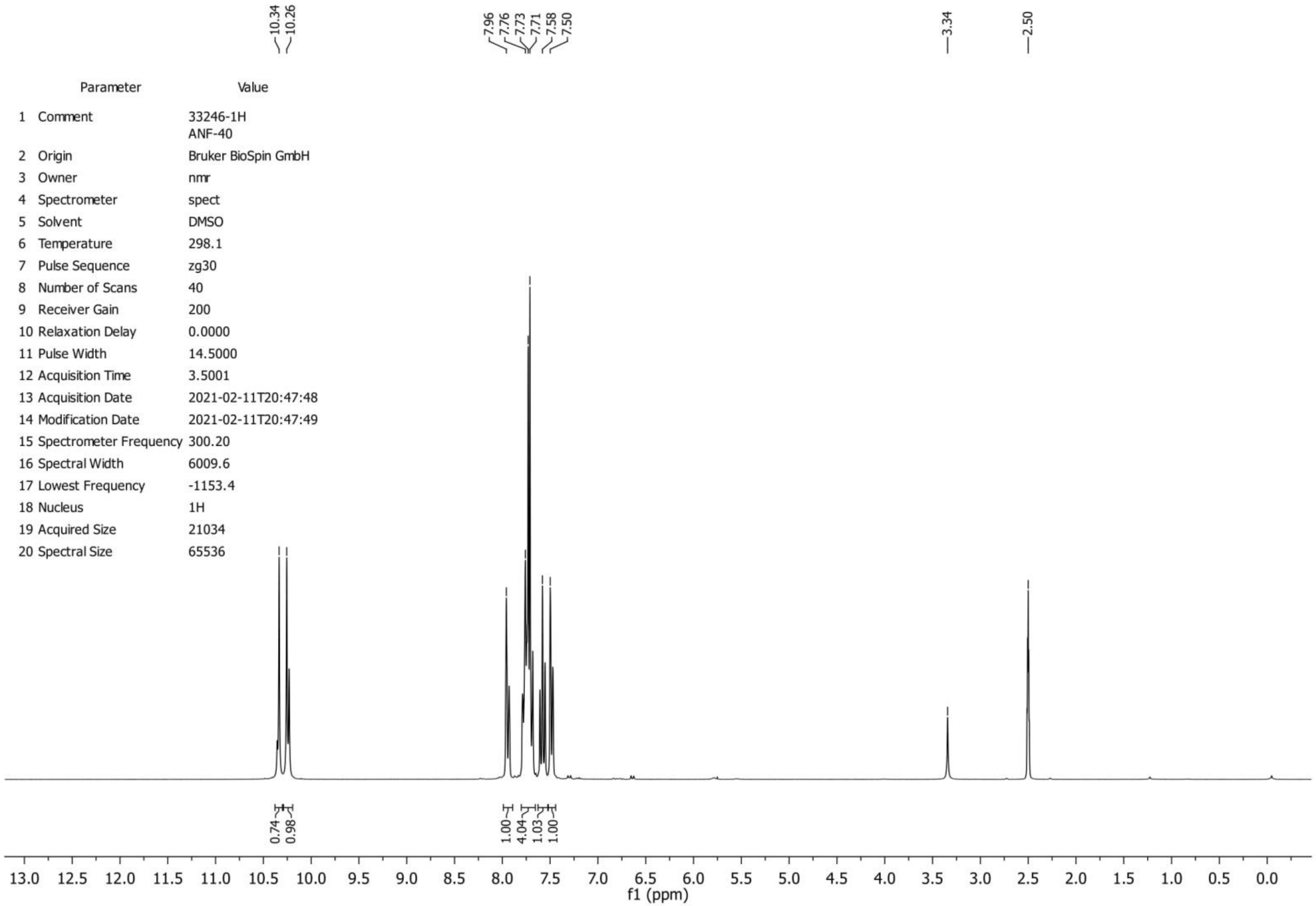

Spectrum 11. ^{1}H NMR of compound **8** (300 MHz, DMSO-d_6).

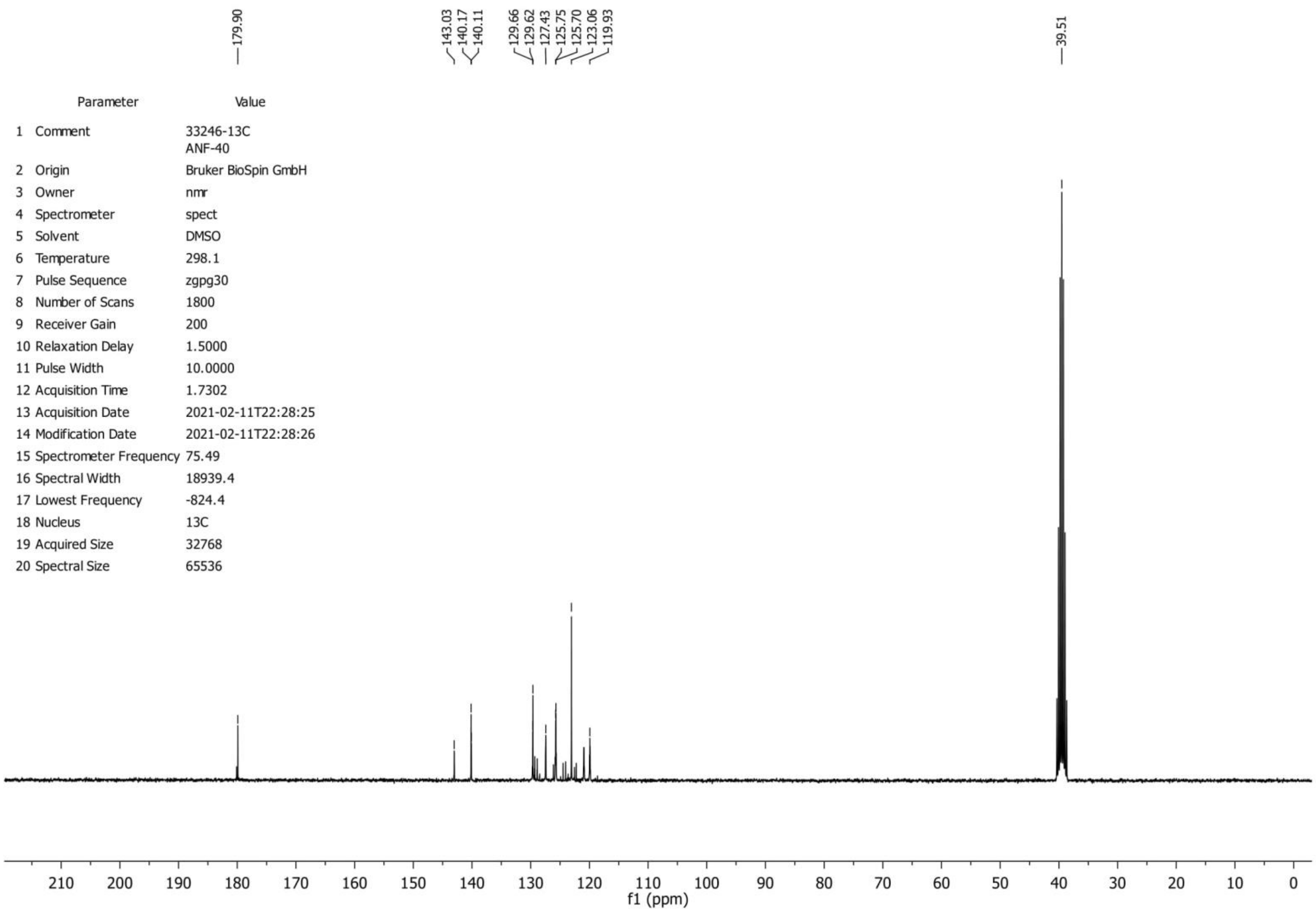

Spectrum 12. ^{13}C NMR of compound **8** (75.5 MHz, DMSO-d_6).

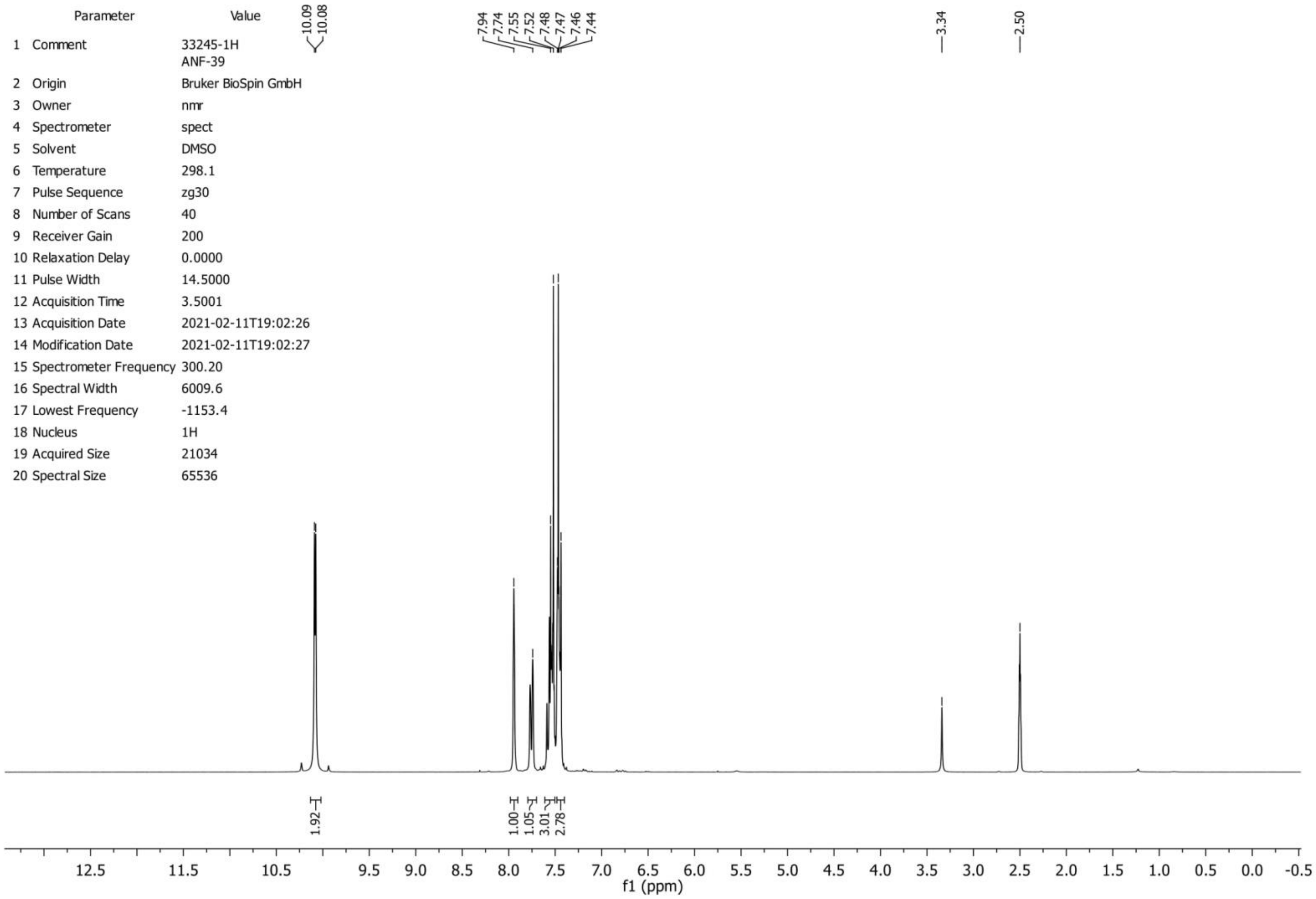

Spectrum 13. ^{1}H NMR of compound **11** (300 MHz, DMSO-d_6).

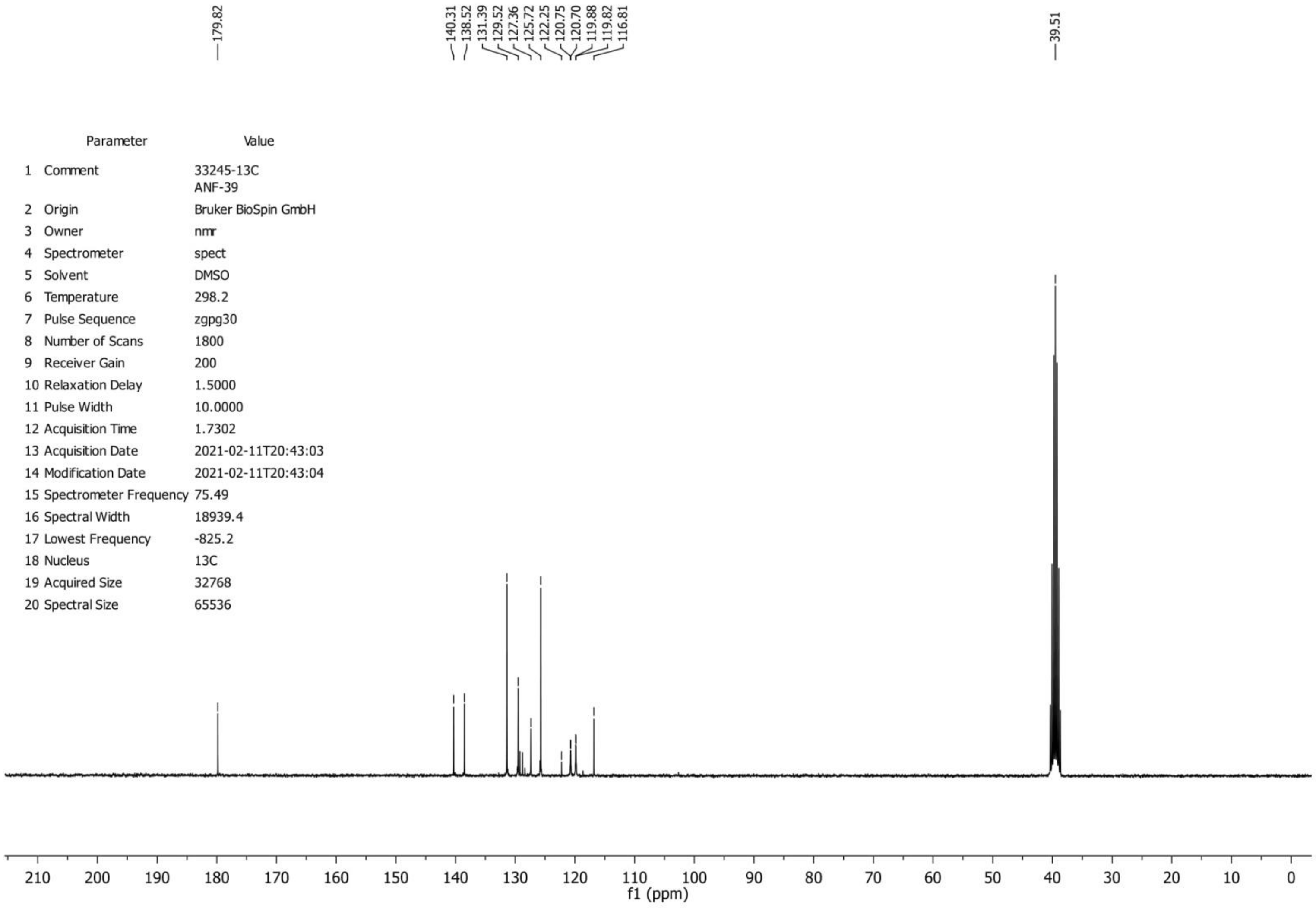

Spectrum 14. ^{13}C NMR of compound **11** (75.5 MHz, DMSO-d_6).

Table S1. High Resolution Mass Spectra (HRMS) of compounds **4, 6, 7, 11**.

compound 4
$[M-H]^-$

Elemental Composition Report
Single Mass Analysis
Tolerance = 5.0 PPM / DBE: min = -0.5, max = 50.0
Monoisotopic Mass, Odd and Even Electron Ions
2252 formula(e) evaluated with 56 results within limits (up to 50 closest results for each mass)

	Mass	Calc. Mass	mDa	PPM	DBE	Formula
Minimum:					-0.5	
Maximum:			200.0	5.0	50.0	
	362.9732	362.9732	0.0	0.0	2.0	C8 H11 N3 O3 F2 Na Cl3
		362.9731	0.0	0.1	2.0	C8 H9 N3 O F5 Cl3
		362.9731	0.0	0.1	3.0	C9 H8 N O3 F5 Na S Cl
		362.9732	-0.1	-0.2	14.0	C17 H5 N O F3 S Cl
		362.9731	0.1	0.2	10.5	C12 H6 N4 O3 F Na S Cl
		362.9731	0.1	0.3	10.0	C15 H9 N F2 Na S2 Cl
		362.9733	-0.1	-0.3	14.0	C17 H7 N O3 Na S Cl
		362.9733	-0.1	-0.3	13.0	C16 H8 N3 O Cl3
		362.9730	0.1	0.3	10.5	C12 H4 N4 O F4 S Cl
		362.9733	-0.2	-0.4	5.5	C13 H10 O F4 Cl3
		362.9734	-0.2	-0.6	5.5	C13 H12 O3 F Na Cl3
		362.9729	0.3	0.8	6.5	C10 H8 N4 F3 Na S2 Cl
		362.9735	-0.4	-1.0	6.0	C9 H7 N5 F4 S Cl2
		362.9728	0.4	1.0	17.5	C19 H5 N2 O2 Cl2
		362.9728	0.4	1.0	8.5	C14 H10 O3 F2 S2 Cl
		362.9736	-0.4	-1.1	6.0	C9 H9 N5 O2 F Na S Cl2
		362.9726	0.5	1.4	6.5	C11 H6 N2 O2 F5 Cl2
		362.9726	0.5	1.5	4.0	C8 H12 N5 O3 S Cl3
		362.9737	-0.6	-1.5	9.5	C14 H8 N2 F3 S Cl2
		362.9726	0.6	1.6	14.0	C14 H4 N5 O2 F Cl2
		362.9738	-0.6	-1.6	10.0	C11 H5 N5 O3 F2 Cl2
		362.9726	0.6	1.6	5.0	C9 H9 N3 O3 F3 S2 Cl
		362.9738	-0.6	-1.7	9.5	C14 H10 N2 O2 Na S Cl2

		362.9738	-0.6	-1.7	2.5	C8 H7 N2 O3 F6 Cl2
		362.9725	0.7	1.9	2.5	C9 H10 N2 O F4 Na S Cl2
		362.9724	0.7	2.0	10.0	C12 H8 N5 O Na S Cl2
		362.9740	-0.8	-2.1	13.5	C16 H6 N2 O3 F Cl2
		362.9740	-0.8	-2.3	2.5	C7 H9 N4 O F4 Na S2 Cl
		362.9741	-0.9	-2.5	4.0	C9 H6 N3 F6 Na Cl2
		362.9722	0.9	2.6	9.5	C16 H11 O2 Na Cl3
		362.9722	1.0	2.7	9.5	C16 H9 F3 Cl3
		362.9742	-1.0	-2.8	6.5	C9 H5 N4 O2 F5 S Cl
		362.9721	1.0	2.8	8.0	C14 H12 N O F S2 Cl2
		362.9742	-1.0	-2.9	6.0	C12 H10 N O F3 Na S2 Cl
		362.9742	-1.1	-2.9	15.0	C17 H5 N3 F Na Cl2
		362.9721	1.1	2.9	18.0	C20 H4 N F2 S Cl
		362.9743	-1.1	-3.0	7.5	C14 H7 F5 Na Cl2
		362.9720	1.1	3.1	6.0	C11 H10 N3 O2 F Na Cl3
		362.9744	-1.2	-3.2	17.5	C17 H4 N4 O2 S Cl
		362.9720	1.2	3.3	6.0	C11 H8 N3 F4 Cl3
		362.9720	1.2	3.3	7.0	C12 H7 N O2 F4 Na S Cl
		362.9744	-1.2	-3.4	10.0	C14 H6 N O2 F4 S Cl
		362.9719	1.2	3.4	14.5	C15 H5 N4 O2 Na S Cl
		362.9719	1.2	3.4	4.5	C9 H11 N4 O F2 S2 Cl2
		362.9744	-1.3	-3.5	9.0	C13 H9 N3 O2 F Cl3
		362.9745	-1.3	-3.6	1.5	C10 H11 O2 F5 Cl3
		362.9718	1.4	3.8	3.5	C7 H6 N4 O2 F5 Na S Cl
		362.9746	-1.5	-4.0	12.5	C18 H10 O2 Cl3
		362.9717	1.5	4.2	12.5	C17 H9 O2 F S2 Cl
		362.9715	1.6	4.5	10.5	C14 H7 N2 O3 F Na Cl2

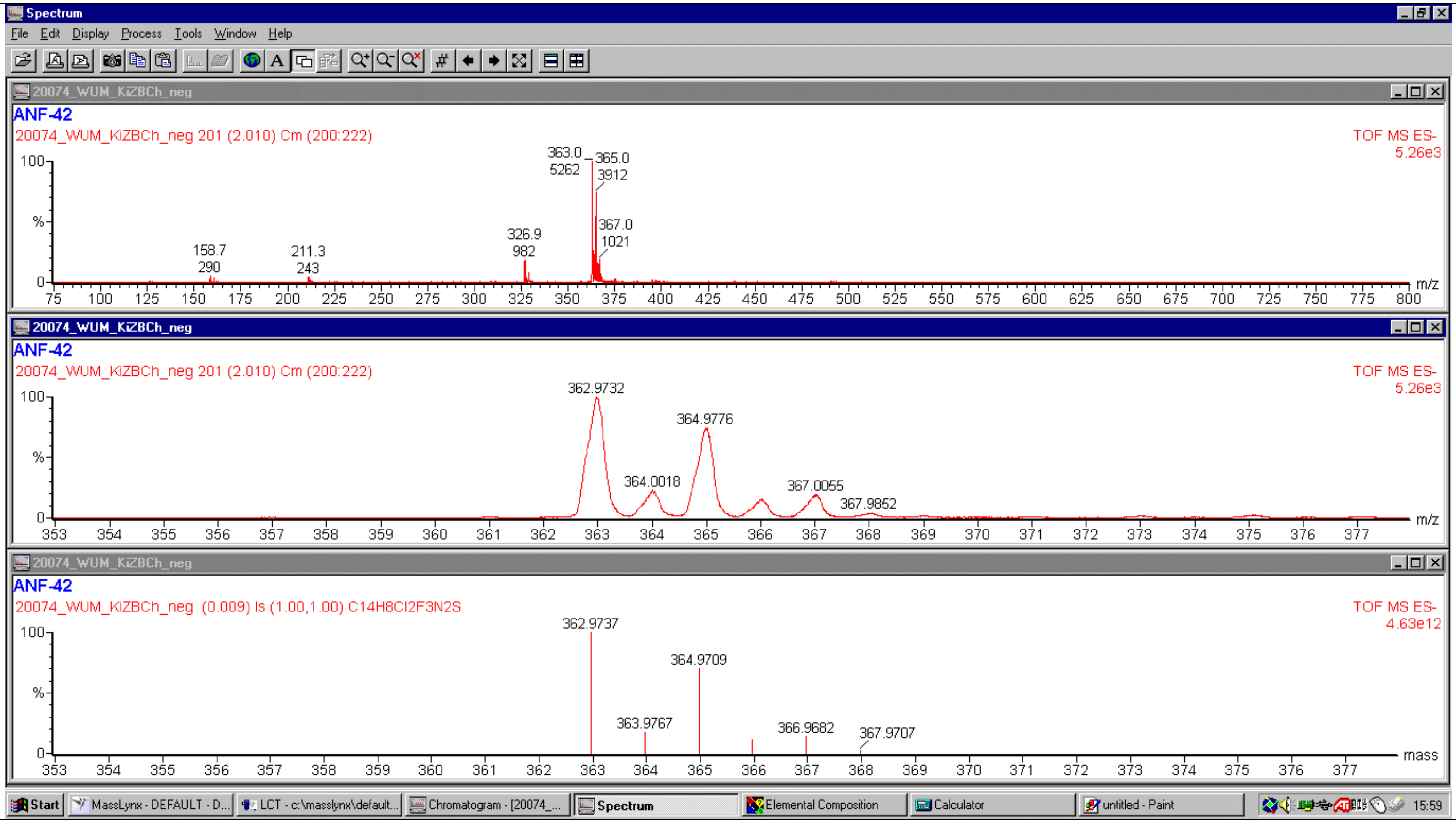

Compound 6
[M-H]$^-$

Single Mass Analysis
Tolerance = 5.0 PPM / DBE: min = -0.5, max = 50.0
Monoisotopic Mass, Odd and Even Electron Ions
2888 formula(e) evaluated with 50 results within limits (up to 50 closest results for each mass)

Mass	Calc. Mass	mDa		PPM	DBE	Formula
Minimum:				-0.5		
Maximum:		200.0	5.0	50.0		
343.0299	343.0300	-0.1	-0.3	1.0		C9 H15 N3 F4 S Cl2
	343.0300	-0.1	-0.3	11.0		C15 H9 N3 O F2 Na Cl

		343.0297	0.1	0.4	8.0	C16 H16 N O Cl3
		343.0300	-0.2	-0.4	1.0	C9 H17 N3 O2 F Na S Cl2
		343.0300	-0.2	-0.5	3.5	C12 H11 O F6 Na Cl
		343.0302	-0.3	-0.9	4.5	C14 H16 F3 S Cl2
		343.0302	-0.3	-0.9	14.5	C20 H10 O F Na Cl
		343.0302	-0.3	-1.0	5.0	C11 H13 N3 O3 F2 Cl2
		343.0295	0.3	1.0	4.5	C11 H15 N4 O F Cl3
		343.0295	0.3	1.0	5.5	C12 H14 N2 O3 F Na S Cl
		343.0295	0.4	1.1	5.5	C12 H12 N2 O F4 S Cl
		343.0295	0.4	1.2	13.0	C15 H10 N5 O S Cl
		343.0304	-0.5	-1.5	8.5	C16 H14 O3 F Cl2
		343.0293	0.5	1.6	2.0	C7 H13 N5 O3 F2 Na S Cl
		343.0304	-0.5	-1.6	5.0	C10 H15 N5 O Na S2 Cl
		343.0293	0.6	1.6	1.5	C10 H16 N2 F3 Na S2 Cl
		343.0293	0.6	1.7	2.0	C7 H11 N5 O F5 S Cl
		343.0305	-0.6	-1.8	6.5	C12 H12 N4 F2 Na Cl2
		343.0293	0.6	1.8	12.5	C19 H13 O2 Cl2
		343.0306	-0.7	-2.1	9.0	C12 H11 N5 O2 F S Cl
		343.0306	-0.8	-2.2	1.5	C9 H13 N2 O2 F5 S Cl
		343.0291	0.8	2.3	1.5	C11 H14 O2 F5 Cl2
		343.0307	-0.8	-2.3	10.0	C17 H13 N F Na Cl2
		343.0307	-0.8	-2.4	0.5	C8 H16 N4 O2 F2 Cl3
		343.0291	0.8	2.4	9.0	C14 H12 N3 O2 F Cl2
		343.0290	0.8	2.4	0.0	C9 H17 N O3 F3 S2 Cl
		343.0290	0.8	2.4	18.5	C23 H9 Na Cl
		343.0308	-0.9	-2.7	12.5	C17 H12 N2 O2 S Cl
		343.0289	1.0	2.9	7.5	C15 H10 F5 Na Cl
		343.0289	1.0	2.9	5.0	C12 H16 N3 O Na S Cl2
		343.0309	-1.0	-3.0	4.0	C13 H17 N O2 F Cl3
		343.0288	1.0	3.0	15.0	C18 H8 N3 F Na Cl
		343.0287	1.2	3.5	4.0	C10 H9 N3 F6 Na Cl
		343.0311	-1.2	-3.5	7.0	C12 H8 N3 F6 Cl
		343.0311	-1.3	-3.7	7.0	C12 H10 N3 O2 F3 Na Cl

	343.0286	1.3	3.8	13.5	C17 H9 N2 O3 F Cl
	343.0313	-1.4	-4.0	18.0	C20 H7 N3 F Cl
	343.0313	-1.4	-4.1	8.0	C14 H15 N3 O S Cl2
	343.0313	-1.4	-4.1	10.5	C17 H9 F5 Cl
	343.0284	1.4	4.2	1.0	C11 H16 N F4 Cl3
	343.0313	-1.5	-4.2	0.5	C11 H17 O F4 S Cl2
	343.0313	-1.5	-4.2	10.5	C17 H11 O2 F2 Na Cl
	343.0284	1.5	4.3	2.5	C9 H10 N2 O3 F6 Cl
	343.0284	1.5	4.3	8.5	C14 H14 N4 Cl3
	343.0284	1.5	4.3	9.5	C15 H13 N2 O2 Na S Cl
	343.0284	1.5	4.4	10.0	C12 H8 N5 O3 F2 Cl
	343.0284	1.5	4.4	9.5	C15 H11 N2 F3 S Cl
	343.0315	-1.7	-4.8	1.0	C7 H14 N5 F4 S2 Cl
	343.0282	1.7	4.9	6.0	C10 H12 N5 O2 F Na S Cl
	343.0316	-1.7	-4.9	1.0	C7 H16 N5 O2 F Na S2 Cl

<table>
<tr><td></td><td>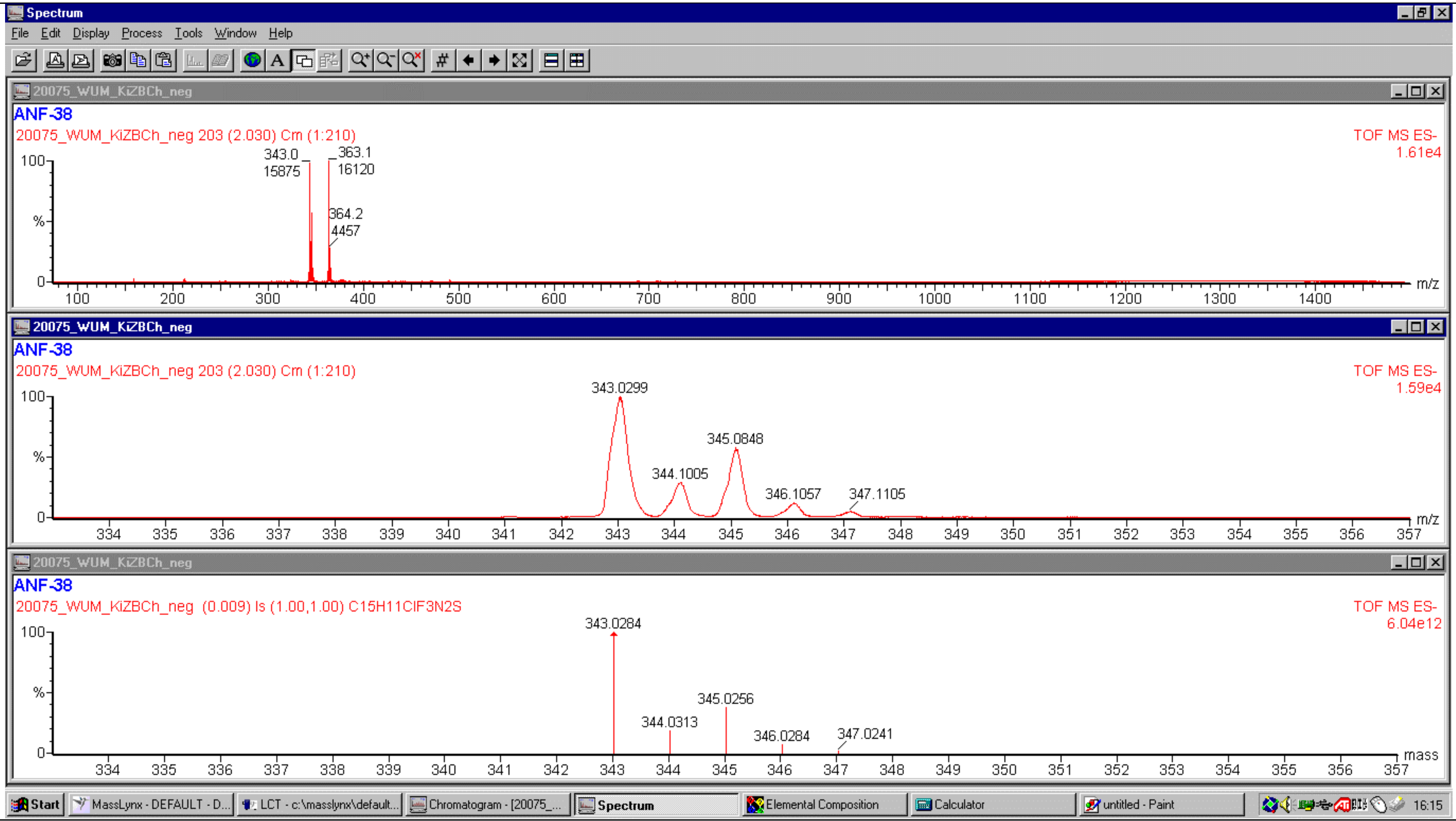
</td></tr>
<tr><td>Compound 7
[M-H]$^{-}$</td><td>Single Mass Analysis
Tolerance = 5.0 PPM / DBE: min = -0.5, max = 50.0
Monoisotopic Mass, Odd and Even Electron Ions
1431 formula(e) evaluated with 33 results within limits (up to 50 closest results for each mass)
Minimum: -0.5
Maximum: 200.0 5.0 50.0</td></tr>
</table>

Mass	Calc. Mass	mDa	PPM	DBE	Formula
363.0379	363.0379	0.0	0.0	14.0	C15 H5 N5 O2 F4
	363.0380	0.0	-0.1	6.5	C12 H7 N2 O2 F8
	363.0378	0.1	0.4	2.5	C10 H11 N2 O F7 Na S
	363.0378	0.2	0.4	3.0	C7 H6 N5 O2 F9
	363.0378	0.2	0.5	10.0	C13 H9 N5 O F3 Na S
	363.0381	-0.2	-0.6	17.5	C20 H6 N2 O2 F3
	363.0375	0.5	1.3	8.0	C15 H13 N O F4 S2
	363.0384	-0.5	-1.3	0.5	C7 H13 N4 O2 F6 S2
	363.0373	0.7	1.8	4.5	C10 H12 N4 O F5 S2
	363.0386	-0.7	-1.9	4.0	C12 H14 N O2 F5 S2
	363.0389	-0.9	-2.6	6.0	C10 H8 N5 F7 S
	363.0389	-1.0	-2.7	6.0	C10 H10 N5 O2 F4 Na S
	363.0369	1.1	2.9	10.5	C15 H8 N2 O3 F4 Na
	363.0368	1.1	3.0	10.5	C15 H6 N2 O F7
	363.0391	-1.1	-3.1	9.5	C15 H9 N2 F6 S
	363.0368	1.1	3.1	18.0	C18 H4 N5 O F3
	363.0391	-1.1	-3.2	10.0	C12 H6 N5 O3 F5
	363.0391	-1.2	-3.2	9.5	C15 H11 N2 O2 F3 Na S
	363.0391	-1.2	-3.3	2.5	C9 H8 N2 O3 F9
	363.0367	1.2	3.4	-0.5	C7 H9 N2 O3 F9 Na
	363.0367	1.3	3.5	7.0	C10 H7 N5 O3 F5 Na
	363.0367	1.3	3.5	6.5	C13 H10 N2 F6 Na S
	363.0366	1.3	3.6	7.0	C10 H5 N5 O F8
	363.0393	-1.3	-3.7	13.5	C17 H7 N2 O3 F4
	363.0394	-1.5	-4.0	4.0	C10 H7 N3 F9 Na
	363.0365	1.5	4.1	3.0	C8 H9 N5 F7 Na S
	363.0364	1.5	4.3	5.0	C12 H11 N O3 F6 S
	363.0363	1.6	4.4	12.0	C18 H12 N F3 S2
	363.0396	-1.6	-4.5	15.0	C18 H6 N3 F4 Na
	363.0396	-1.7	-4.6	7.5	C15 H8 F8 Na
	363.0362	1.7	4.8	1.5	C7 H10 N4 O3 F7 S
	363.0362	1.8	4.9	1.0	C10 H13 N F8 S2

363.0361 1.8 5.0 8.5 C13 H11 N4 F4 S2

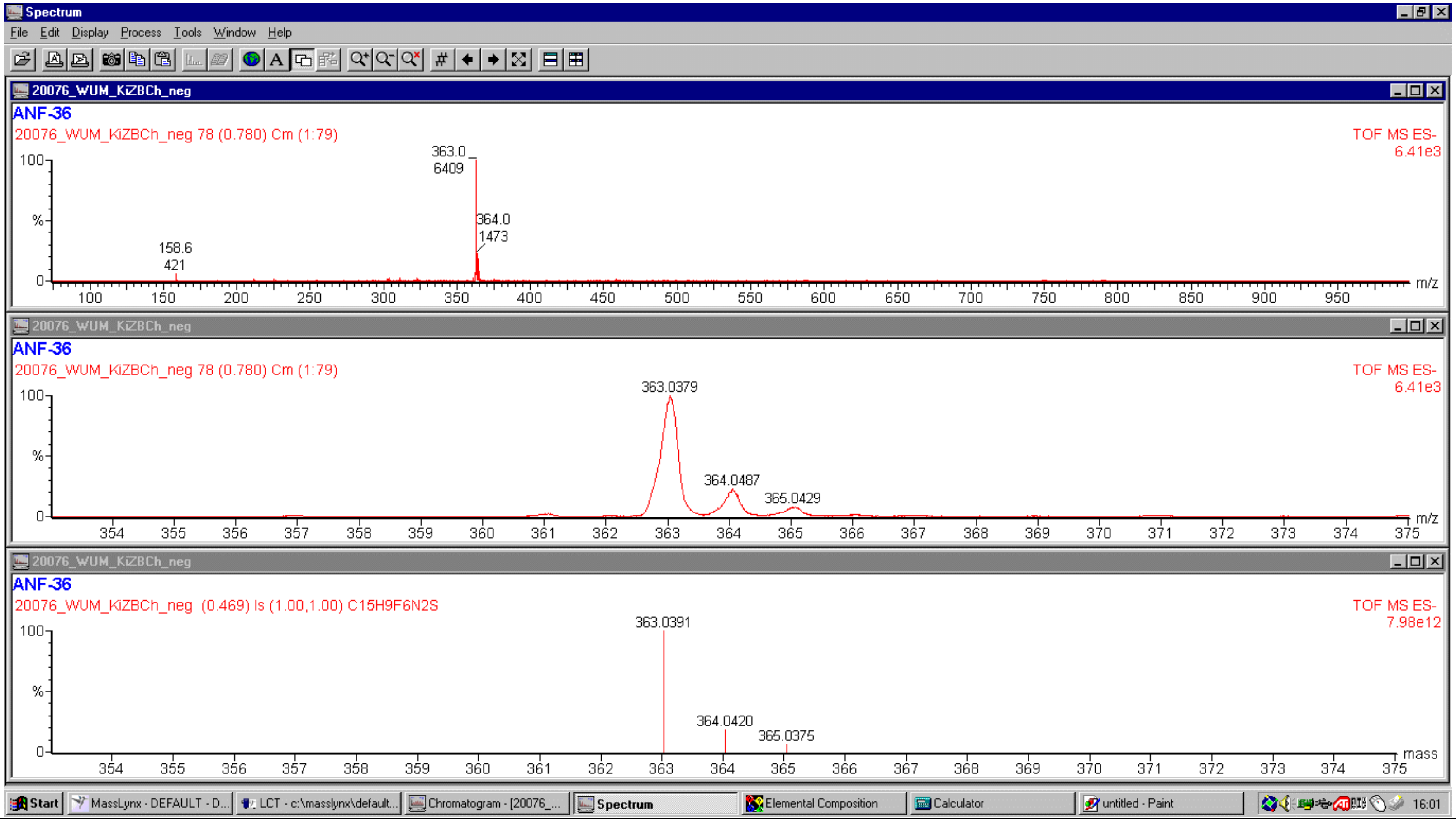

Compound 11
[M-H]$^{-}$

Single Mass Analysis
Tolerance = 5.0 PPM / DBE: min = -0.5, max = 50.0
Monoisotopic Mass, Odd and Even Electron Ions
1398 formula(e) evaluated with 24 results within limits (up to 50 closest results for each mass)
Minimum: -0.5
Maximum: 200.0 5.0 50.0
Mass Calc. Mass mDa PPM DBE Formula

	372.9631	372.9632	0.0	-0.1	1.5	C9 H14 N2 F3 Na S2 Br
		372.9633	-0.2	-0.4	13.0	C14 H8 N5 O S Br
		372.9633	-0.2	-0.5	5.5	C11 H10 N2 O F4 S Br
		372.9634	-0.2	-0.6	5.5	C11 H12 N2 O3 F Na S Br
		372.9627	0.4	1.1	7.5	C14 H8 F5 Na Br
		372.9627	0.5	1.2	15.0	C17 H6 N3 F Na Br
		372.9625	0.6	1.6	4.0	C9 H7 N3 F6 Na Br
		372.9638	-0.7	-1.8	11.0	C14 H7 N3 O F2 Na Br
		372.9624	0.7	2.0	13.5	C16 H7 N2 O3 F Br
		372.9639	-0.7	-2.0	3.5	C11 H9 O F6 Na Br
		372.9622	0.9	2.4	2.5	C8 H8 N2 O3 F6 Br
		372.9640	-0.9	-2.4	14.5	C19 H8 O F Na Br
		372.9622	0.9	2.4	9.5	C14 H11 N2 O2 Na S Br
		372.9622	0.9	2.5	10.0	C11 H6 N5 O3 F2 Br
		372.9622	0.9	2.5	9.5	C14 H9 N2 F3 S Br
		372.9620	1.1	3.0	6.0	C9 H10 N5 O2 F Na S Br
		372.9643	-1.1	-3.0	5.0	C9 H13 N5 O Na S2 Br
		372.9620	1.1	3.1	6.0	C9 H8 N5 F4 S Br
		372.9644	-1.3	-3.5	9.0	C11 H9 N5 O2 F S Br
		372.9645	-1.3	-3.6	1.5	C8 H11 N2 O2 F5 S Br
		372.9617	1.4	3.7	4.0	C11 H14 N O2 F2 S2 Br
		372.9646	-1.5	-4.0	12.5	C16 H10 N2 O2 S Br
		372.9649	-1.8	-4.8	7.0	C11 H6 N3 F6 Br
		372.9650	-1.8	-4.9	7.0	C11 H8 N3 O2 F3 Na Br

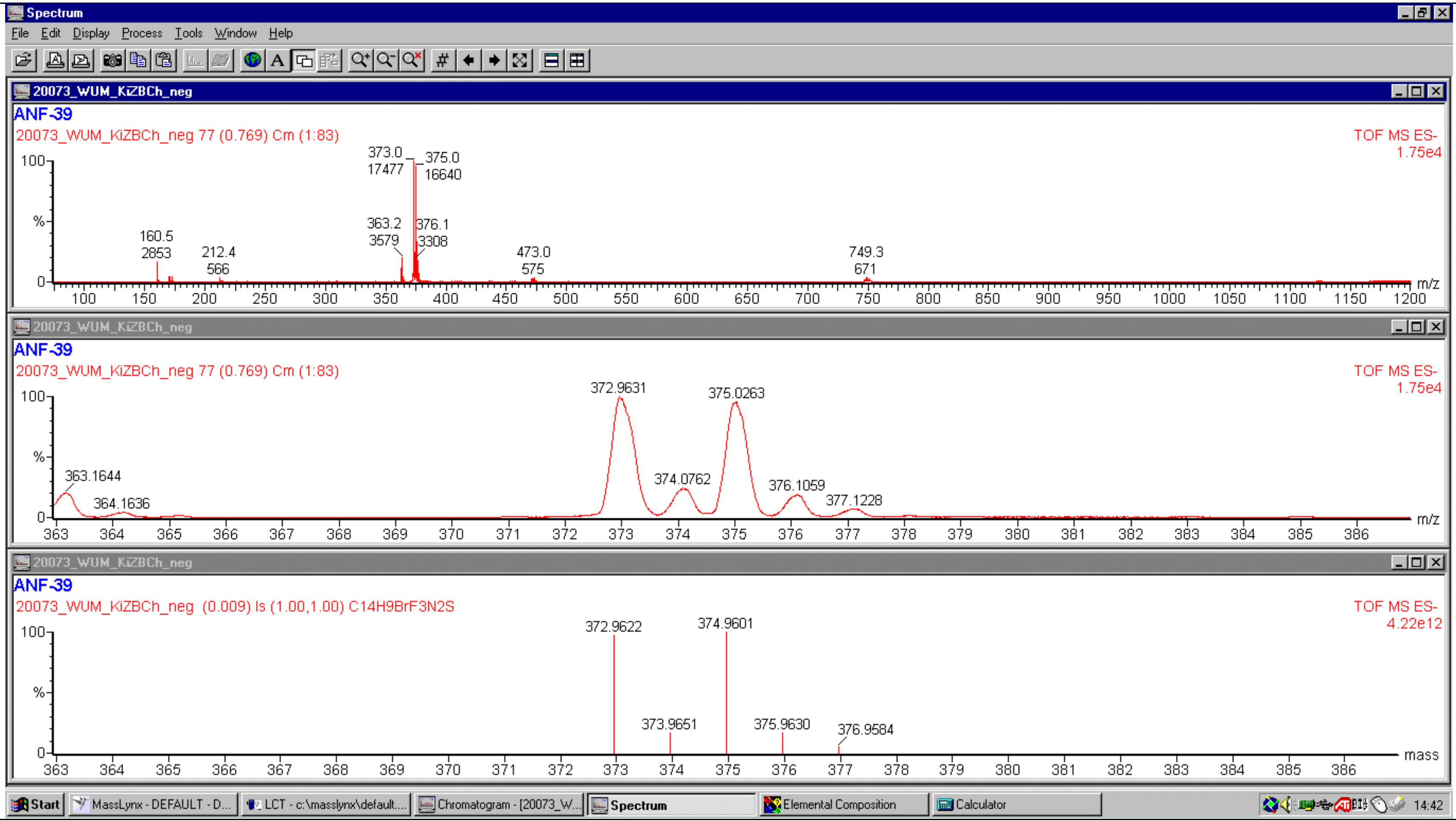
Spectrum
File Edit Display Process Tools Window Help
20073_WUM_KiZBCh_neg
ANF-39
20073_WUM_KiZBCh_neg 77 (0.769) Cm (1:83)
TOF MS ES-
1.75e4
373.0 17477
375.0 16640
363.2 3579
376.1 3308
160.5 2853
212.4 566
473.0 575
749.3 671
372.9631
375.0263
363.1644
364.1636
374.0762
376.1059
377.1228
20073_WUM_KiZBCh_neg (0.009) Is (1.00,1.00) C14H9BrF3N2S
4.22e12
372.9622
374.9601
373.9651
375.9630
376.9584
m/z
mass
Start
MassLynx - DEFAULT - D...
LCT - c:\masslynx\default....
Chromatogram - [20073_W...
Elemental Composition
Calculator
14:42
